# Ultrasound‐Activated Biodegradable Piezoelectric Chitosan Nanoparticles for Glioblastoma Treatment

**DOI:** 10.1002/smsc.202500457

**Published:** 2025-11-21

**Authors:** Attilio Marino, Tommaso Curiale, Marie Celine Lefevre, Alessio Carmignani, Maria Cristina Ceccarelli, Matteo Battaglini, Kamil Ziaja, Sergio Marras, Bruno Torre, Pietro Fiaschi, Gianni Ciofani

**Affiliations:** ^1^ Istituto Italiano di Tecnologia Smart Bio‐Interfaces Viale Rinaldo Piaggio 34 56025 Pontedera Italy; ^2^ Scuola Superiore Sant’Anna The Biorobotics Institute Viale Rinaldo Piaggio 34 56025 Pontedera Italy; ^3^ Department of Chemistry CICECO‐Aveiro Institute of Materials University of Aveiro Rua de Calouste Gulbenkian 1 3810‐074 Aveiro Portugal; ^4^ Nanochemistry Department Istituto Italiano di Tecnologia Via Morego 30 16130 Genova Italy; ^5^ Istituto Nazionale Di Ricerca Metrologica Strada delle Cacce 91 10135 Torino Italy; ^6^ Department of Neurosurgery IRCCS Ospedale Policlinico San Martino Largo Rossana Benzi 10 16132 Genova Italy; ^7^ Department of Neuroscience, Rehabilitation, Ophthalmology, Genetics, Maternal and Child Health (DiNOGMI) University of Genova Largo Paolo Daneo 3 16132 Genova Italy

**Keywords:** biodegradable piezoelectric polymers, bioelectric stimulation, brain cancer, piezoelectric nanoparticles, sonodynamic therapy

## Abstract

Piezoelectric nanomaterials are highly promising for remote cell stimulation due to their ability to convert mechanical energy, such as ultrasound (US), into electrical cues that modulate cellular behavior. In the context of cancer treatment, piezoelectric stimulation has recently shown antiproliferative, chemosensitizing, antiangiogenic, and immunomodulatory effects. Despite growing interest in organic alternatives, no biodegradable or bioabsorbable nanoparticles with clinically approved components have yet been developed with piezoelectric properties for cell stimulation, limiting the translational potential of this approach. Here, chitosan nanoparticles (ChNPs) have been engineered to exhibit intrinsic piezoelectric properties, enabling US‐mediated activation. Their structural, mechanical, and piezoelectric characteristics have been investigated using advanced physicochemical and electromechanical techniques. Biological evaluation of US‐driven ChNPs‐assisted piezostimulation has been tested on patient‐derived glioblastoma cells. When stimulated with US, ChNPs demonstrate not only excellent antiproliferative activity, but also proapoptotic efficacy, even in the absence of any chemotherapeutic agent. This drug‐free anticancer stimulation approach is attributed to reactive oxygen species generation triggered by the ChNP piezocatalytic properties. The antitumor activity is further validated in more complex ex ovo models. The combination of piezoelectric responsiveness, biodegradability, and preclinical feasibility highlights the potential of ChNPs as a safe, noninvasive therapeutic platform for next‐generation cancer treatments.

## Graphical abstract

Biodegradable chitosan nanoparticles are engineered to exhibit intrinsic piezoelectric properties, enabling ultrasound (US) activation for glioblastoma treatment. Their electromechanical behavior, biocompatibility, and anticancer efficacy are demonstrated, showing that US‐driven piezostimulation induces oxidative stress and apoptosis without drugs, offering a noninvasive, bioelectronic therapeutic approach.

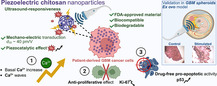

## 1 Introduction

Glioblastoma (GBM) is the most common and lethal primary brain tumor in adults, characterized by pronounced cellular heterogeneity and marked resistance to chemotherapeutic agents.^[^ ^1^, ^2^, ^3^ ^]^ This intrinsic heterogeneity contributes to the poor sensitivity of GBM to conventional treatments and underlies the frequent tumor recurrence that occurs despite standard‐of‐care therapies. Current treatment protocols combine maximal safe surgical resection with radiotherapy and chemotherapy using temozolomide, but these approaches have only modestly improved survival and fail to prevent relapse.^[^ ^4^, ^5^, ^6^, ^7^, ^8^ ^]^ The complex biology of GBM, including the presence of glioma stem‐like cells, invasive behavior, and adaptive tumor microenvironment, presents formidable barriers to effective therapy.

Given these challenges, there is an urgent need for novel, noninvasive physical stimulation strategies capable of selectively inducing tumor cytotoxicity.^[^ ^7^, ^9^ ^]^ Electrical stimulation has been food and drug administration‐approved for GBM treatment and shows promise in modulating tumor growth^[^ ^10^, ^11^, ^12^ ^]^; however, electric fields lack spatial specificity and can inhibit the proliferation of healthy brain cells such as astrocytes, highlighting the necessity for more precise delivery of electrical cues.^[^ ^12^ ^]^

In this context, piezoelectric stimulation has emerged as a promising alternative by converting externally applied mechanical energy, typically ultrasound (US), into localized electrical signals within the tumor microenvironment.^[^ ^9^, ^13^, ^14^, ^15^, ^16^, ^17^, ^18^ ^]^ This method enables spatially controlled modulation of cancer cells while minimizing off‐target effects.^[^ ^17^, ^19^, ^20^ ^]^ Specifically, chronic piezoelectric stimulation in cancer cells is known to disrupt the homeostasis of key ions involved in cell cycle regulation (i.e., calcium and potassium), resulting in cell cycle arrest;^[^ ^19^, ^21^ ^]^ in parallel, it has also been shown to enhance sensitivity to chemotherapeutic agents.^[^ ^20^ ^]^ Piezostimulation thus reproduces the biological effects of low‐intensity electric fields, but with the added advantage of spatial precision and remote activation via US, increasing its therapeutic applicability. Moreover, recent technological advances, including MRI‐guided focused US systems^[^ ^9^, ^22^ ^]^ and localized brain cancer infusion techniques following surgical resection,^[^ ^23^ ^]^ provide a promising foundation for the future clinical development of site‐specific delivery and remote activation of piezoelectric nanoparticles in the brain, enabling noninvasive and spatially controlled GBM therapy.^[^ ^19^, ^24^ ^]^

Among inorganic piezoelectric materials, barium titanate (BaTiO_3_) nanoparticles have been extensively studied due to their high piezoelectric coefficients, chemical stability, and relative biocompatibility. When activated by external US stimulation, BaTiO_3_ nanoparticles produce transient electrical signals that can perturb cancer cell membranes, disrupt intracellular signaling, and induce oxidative damage via the formation of reactive oxygen species (ROS). Several studies have demonstrated that BaTiO_3_‐based piezoelectric systems can inhibit tumor growth, sensitize GBM cells to chemotherapy, and overcome limitations associated with drug resistance and the presence of the blood–brain barrier.^[^ ^18^, ^19^, ^21^, ^24^, ^25^, ^26^ ^]^ Organic piezoelectric materials like poly(vinylidene fluoride)‐PVDF and its copolymer poly(vinylidene fluoride‐trifluoroethylene)‐P(VDF‐TrFE) have gained attention for biomedical applications due to biocompatibility and tunable crystallinity.^[^ ^26^, ^27^, ^28^, ^29^, ^30^, ^31^ ^]^ Their piezoelectric activity arises from the crystalline β‐phase, enabling effective dipole alignment.^[^ ^31^ ^]^ Upon US stimulation, P(VDF‐TrFE)‐based nanoparticles can exert antiproliferative effects on cancer cells and enhance their sensitivity to chemotherapeutic agents.^[^ ^20^ ^]^ However, the synthetic nature and poor biodegradability of this polymer limit its clinical translatability.

In this scenario, we propose the use of chitosan‐based piezoelectric nanoparticles (ChNPs) as a sustainable and biodegradable alternative for piezoelectric anticancer therapy. Chitosan is a natural polysaccharide derived from chitin, the second most abundant biopolymer in nature. It is widely recognized for its excellent biocompatibility, low immunogenicity, and intrinsic bioactivity, including antimicrobial and wound‐healing properties. Chitosan polycationic structure allows for facile chemical modification and interaction with negatively charged cellular membranes, making it highly suitable for biomedical applications, particularly in drug delivery and tissue engineering.^[^ ^18^, ^32^, ^33^, ^34^, ^35^, ^36^, ^37^, ^38^, ^39^ ^]^ Recent studies on chitosan films have demonstrated piezoelectric properties, attributed to their semicrystalline structure and to the alignment of dipolar amine groups.^[^ ^40^, ^41^ ^]^ However, such piezoelectric behavior has been observed exclusively in film configurations.^[^ ^41^, ^42^ ^]^

In this study, we report the first‐ever design, synthesis, and characterization of piezoelectric chitosan ChNPs, specifically engineered for biomedical applications. To the best of our knowledge, this is the first work to successfully develop and apply chitosan‐based nanoparticles with intrinsic piezoelectric properties, according to a recently patented procedure.^[^ ^43^ ^]^ ChNPs were fabricated using an optimized emulsification technique, resulting in stable, uniform nanostructures with strong piezoelectric features, higher than PVDF‐TrFE nanoparticles^[^ ^28^, ^29^, ^31^, ^44^ ^]^ and comparable to those of inorganic piezoelectric nanoparticles (i.e., BaTiO_3_).^[^ ^45^ ^]^ To assess their therapeutic potential, we applied chronic US‐driven stimulation to patient‐derived GBM cells. Remarkably, ChNPs induced significant antiproliferative and proapoptotic effects even in the absence of chemotherapeutic agents, highlighting a drug‐free anticancer mechanism. Our investigations revealed the generation of reactive ROS, including singlet oxygen, consistent with a piezocatalytic process. Antitumor efficacy was further validated in complex 3D patient‐derived spheroids implanted in ovo. Collectively, these findings support the potential of ChNPs as a noninvasive, remotely activatable platform for targeted treatment of aggressive tumors such as GBM.

## 2 Results and Discussion

### 2.1 Preparation and Characterization of ChNPs

**Figure 1:**
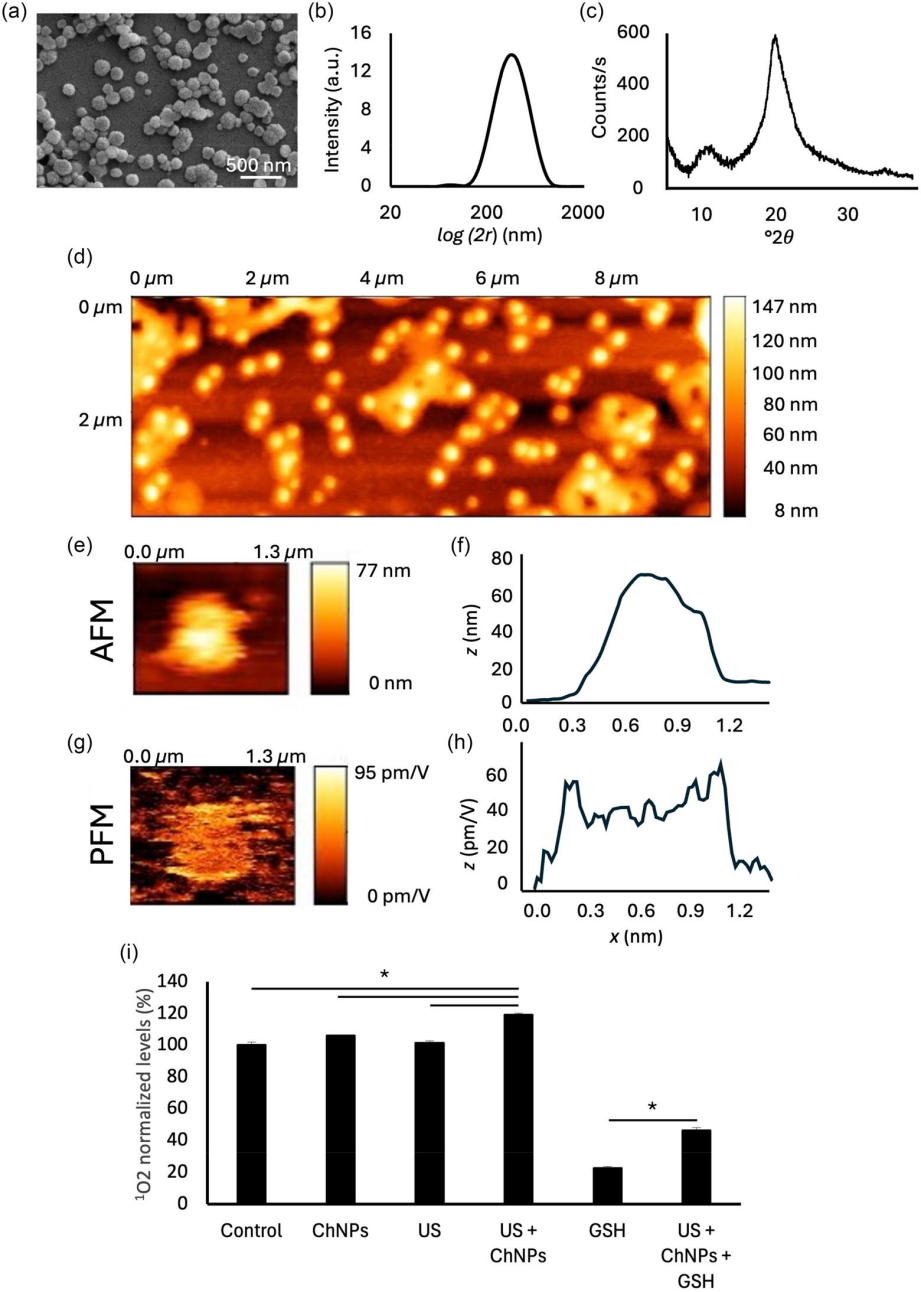
ChNP characterization. a) SEM imaging, b) DLS analysis, c) XRD analysis, d) topographic map of the chitosan nanoparticles in AFM mode (without voltage application), e) topographic map of a single chitosan nanoparticle with f) the respective profilometric graph obtained in AFM mode, g) PFM of the same nanoparticle with h) the respective PFM profile showing the displacement in response to electrical stimulation. i) Piezocatalytic activity in PBS (Control, ChNPs, US, and US + ChNPs) and in PBS with GSH (with a concentration of 10 mM, GSH and US + ChNPs + GSH) (*p < 0.001).

ChNPs were fabricated using an optimized water‐in‐oil emulsification method, specifically designed to endow the particles with piezoelectric properties (Figure S1, Supporting Information). This approach, where potassium hydroxide (KOH) works as a key structuring agent, was developed to overcome the intrinsic lack of piezoelectricity in conventionally prepared ChNPs by ionotropic gelation, and to enable their use as mechanoresponsive agents for biomedical applications.^[^ ^43^ ^]^ The overall yield of the synthesis, as determined through weight measurement after freeze drying, was ≈42%, which is in line with the yields commonly reported for ChNPs prepared via emulsification and ionotropic gelation.^[^ ^35^, ^46^ ^]^ Morphology, size distribution, structural properties, piezoelectric response, and piezocatalytic properties of the ChNPs were investigated (**Figure** **1**).

Scanning electron microscopy (SEM) imaging (Figure 1a) revealed that the ChNPs exhibit a spherical‐like morphology; dynamic light scattering (DLS) analyses reported a hydrodynamic diameter of 362 ± 15 nm (Figure 1b), a ζ‐potential of +8.3 ± 4.1 mV, and a polydispersity index (PDI) of 0.284 ± 0.035, indicating a moderately monodisperse distribution.^[^ ^47^ ^]^ ChNPs exhibit a positive ζ‐potential due to the chitosan chemical structure: indeed, chitosan is a linear polysaccharide consisting of repeating units of glucosamine and *N*‐acetylglucosamine; the glucosamine units contain amine groups (‐NH_2_) that are protonated under acidic to slightly neutral pH conditions.^[^ ^32^, ^34^, ^36^ ^]^ X‐ray diffraction (XRD) analysis (Figure 1c) demonstrated a broad peak centered at *2θ* = 20°, consistent with the semicrystalline nature of chitosan.^[^ ^38^, ^48^ ^]^ The broadness of this peak suggests the presence of short‐range molecular order (i.e., semicrystallinity) rather than complete crystallinity, a structural configuration that supports local dipole formation and alignment. This intermediate ordering is particularly favorable for piezoelectric activity in polymers, as it balances mechanical flexibility with the molecular asymmetry required to generate an electric response under stress.^[^ ^49^, ^50^ ^]^

Figure 1d displays the atomic force microscopy (AFM) topographic map of a large area populated with nanoparticles deposited on a silica substrate, revealing their spherical shape and uniform distribution. A high‐resolution scan of an individual representative nanoparticle is shown in Figure 1e, with the corresponding height profile reported in Figure 1f. Piezoresponse force microscopy (PFM) analysis on the respective nanoparticle is shown in Figure 1g, which highlights a measurable electromechanical response localized over the particle surface. The corresponding amplitude line scan is shown in Figure 1h. The average *d*_33_ coefficient of ChNPs was found to be 37.3 ± 4.5 pm V^−1^ (*n* = 15).

The observed piezoelectric performance of KOH‐structured ChNPs represents a significant achievement. While piezoelectric chitosan composites have been previously reported,^[^ ^18^, ^36^ ^]^ this study is the first to demonstrate intrinsic piezoelectricity in nanoparticles composed solely of chitosan. PFM measurements revealed a *d*_33_ substantially higher than that of bulk chitosan films (7–15 pm V^−1^^[^ ^42^ ^]^). Moreover, this response exceeds that of well‐known piezoelectric polymers such as P(VDF‐TrFE), which typically exhibit *d*_33_ values in the range of 20–30 pm V^−1^ in nanoparticle form.^[^ ^28^, ^29^, ^31^, ^44^ ^]^ Notably, the piezoelectric performance of obtained ChNPs is also comparable to certain inorganic piezoelectric nanoparticles, for example BaTiO_3_ nanoparticles (≈30 pm V^−1^^[^ ^45^ ^]^).

**Table** **1** summarizes the main piezoelectric nanotransducers reported in the literature, with a focus on their nature (organic or inorganic), size, piezoelectric properties (*d*_33_ coefficient), and biodegradability.

**Table 1:** Overview of representative piezoelectric nanotransducers reported in the literature for biomedical applications, highlighting their chemical nature (organic or inorganic), size, piezoelectric coefficient, and biodegradability.

Type	Material	Size	Piezoelectric coefficient (*d*_33_)	Biocompatibility	Biodegradability	Ref.
Inorganic	BaTiO_3_ nanoparticles	100–500 nm	≈30 pm V^−1^	Yes	No	[45]
	ZnO nanorods	150–500 nm (diameter) 400–1500 nm (length)	≈0.5–15 pm V^−1^	Partially	No	[78, 79, 80]
	ZnO nanobelts	65 nm–thick 360 nm wide Tens of μm (length)	14.3‐26.7 pm V^−1^	Partially	No	[81]
	Boron nitride nanotubes	≈50–120 nm (diameter) ≈30 μm (length)	31.2 pm V^−1^ (*d*_31_)	Partially	No	[82]
Organic	P(VDF‐TrFE) nanoparticles	≈200 nm	20–30 pm V^−1^	Yes	No	[28, 29, 31, 44]
	Nylon‐11 nanowires	≈200 nm (diameter) hundreds of μm (length)	3.22 pm V^−1^	Yes	No	[46]
	ChNPs	362 ± 15 nm	37 ± 4 pm V^−1^	Yes	Yes	Present work

The emergence of nanoscale piezoelectricity in ChNPs can likely be attributed to the alkaline conditions introduced by KOH during the emulsification process. This is in line with previous studies showing that alkaline precipitation processes, such as NaOH or KOH treatment, increase chitosan crystallinity via a dissolution–reprecipitation mechanism.^[^ ^51^ ^]^ In order to confirm this hypothesis, chitosan nanoparticles obtained by ionotropic gelation without KOH, indicated as ChNPs* were synthesized to evaluate the impact of KOH on the piezoelectric behavior. Morphological analysis by SEM (Figure S2a, Supporting Information) revealed well‐formed, spherical nanoparticles, indicating that KOH is not essential for particle formation. DLS measurements (Figure S2b, Supporting Information) showed an average size of 431 ± 12 nm and a PDI of 0.289 ± 0.004. As hypothesized, AFM/PFM analysis (Figure S2c‐e, Supporting Information) did not detect any measurable piezoelectric response. This result supports the hypothesis that the presence of KOH during synthesis is crucial for promoting the piezoelectric behavior, likely due to its role in rearranging the chitosan chains into more ordered structures.^[^ ^51^ ^]^

Eventually, piezocatalytic properties of ChNPs were investigated (Figure 1i). Following a single US stimulation session (1 h) in the presence of ChNPs (US + ChNPs), a significant increase in singlet oxygen (^1^O_2_) levels was observed, corresponding to a 20% overproduction compared to the control (*p* < 0.001). This increase in ROS production indicates that the ChNPs effectively transduce mechanical energy into biochemical signaling through the piezoelectric effect; conversely, control groups treated with ChNPs alone or US alone showed no appreciable change in ^1^O_2_ levels compared to untreated samples. The use of appropriate controls (no particles, US only, ChNPs only) confirmed that no significant background fluorescence or spectral interference occurs: the observed fluorescence increment is therefore specifically induced by the combination of US and ChNPs, reliably reporting ^1^O_2_ generation.

These results confirm the functional relevance of the piezoelectric response: only when mechanical stimulation via US is applied in the presence of piezoelectric ChNPs a measurable oxidative output does emerge, likely mediated by electrochemical redox activation at the nanoparticle surface.

To simulate the reductive conditions of the tumor microenvironment, additional experiments were furthermore conducted in the presence of reduced glutathione (GSH, 10 mM). Under these conditions, piezoelectric stimulation (US + ChNPs + GSH) still led to a significant enhancement in ^1^O_2_ generation, corresponding to an about 25% increment relative to samples containing GSH alone. This finding demonstrates that the piezoelectric response of ChNPs remains functionally active even in a glutathione‐rich environment, highlighting their robustness and potential applicability within the oxidative‐reductive balance typical of cancer tissues.^[^ ^52^ ^]^

Representative emission spectra related to experiments summarized in Figure 1i are reported in Figure S3, Supporting Information.

**Figure 2:**
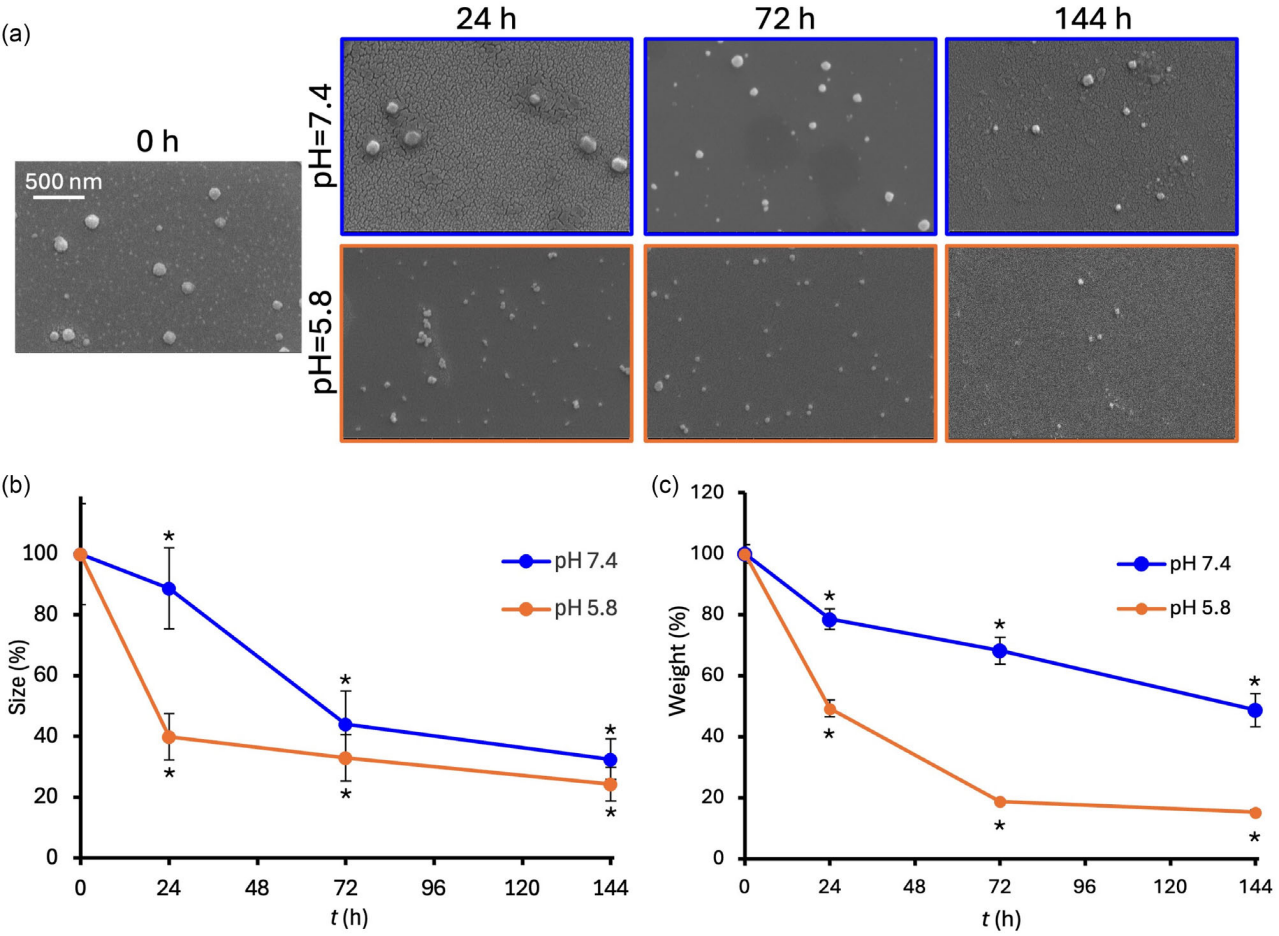
Degradation of ChNPs. a) Representative SEM images of ChNPs acquired at various time points under two different degradation conditions: physiological pH 7.4 (top row, highlighted in blue) and acidic pH 5.8 in the presence of H_2_O_2_ (bottom row, highlighted in orange). Graphs showing the degradation of ChNPs, expressed as change in particle b) diameter and c) weight, under the two experimental conditions (*p < 0.001 with respect to *t* = 0 h).

The stability and degradation behavior of the synthesized ChNPs were assessed over time under both physiological and tumor‐like acidic conditions (**Figure** **2**). ChNPs were incubated in phosphate buffered saline (PBS) at pH 7.4 or in a mildly acidic PBS solution (pH 5.8) supplemented with hydrogen peroxide (200 μM) to mimic the oxidative stress characteristic of the tumor microenvironment. As shown in Figure 2a, SEM images show the progressive degradation of ChNPs under both pH conditions; at *t* = 0, SEM analysis indicated an average nanoparticle diameter of 125 ± 20 nm, which is lower than the corresponding DLS measurement due to hydration layers and partial aggregation effects. At physiological pH (7.4), the nanoparticles initially preserved their spherical morphology, but signs of size reduction became evident at 72 h, becoming more pronounced at 144 h. Under mildly acidic conditions (pH 5.8), ChNPs exhibited earlier and more severe degradation. Figure 2b presents the normalized particle size over the course of the experiment. A sharp decline in diameter was observed under acidic conditions, with particles retaining only 24 ± 6% of their initial size at 144 h. In comparison, ChNPs in neutral pH were 32 ± 7% of their original diameter, confirming a degradation profile more pronounced under tumor‐mimicking environments. To further corroborate the degradation behavior, the mass loss of ChNPs was monitored over time using UV–vis spectroscopy (Figure 2c), showing a rapid loss of particle mass under acidic conditions, with only 15 ± 1% of the original mass remaining at 144 h, while 49 ± 5% of the nanoparticle mass was still present in neutral pH condition at the same time point (144 h), confirming a pH‐dependent degradation behavior.

These findings demonstrate that the ChNPs possess a pH‐sensitive degradation profile, as seen in the literature for other chitosan‐based nanocomposites.^[^ ^32^, ^40^ ^]^ While their gradual disintegration under acidic and oxidative conditions may reduce long‐term piezoelectric responsiveness, this property can be strategically exploited for combined therapeutic functions. In particular, the observed degradation in tumor‐like environments suggests that these nanoparticles could serve not only as piezoelectric transducers, but also as pH‐responsive drug carriers. This could enable a two‐phase treatment strategy: initial electromechanical stimulation followed by local drug delivery, enhancing overall therapeutic efficacy.

### 2.2 Cytocompatibility and Internalization of ChNPs

Biocompatibility of ChNPs on both patient‐derived GBM cells and primary human astrocytes at 72 h of exposure to increasing concentrations of ChNPs (100–600 μg mL^−1^) is reported in Figures S4 and S5, Supporting Information, respectively. In both cell types, no significant reduction in viability was observed across the tested concentrations: these consistent results across both cancerous and healthy human neural cells confirmed the excellent biocompatibility of ChNPs. Obtained data confirm that ChNPs are well tolerated by both patient‐derived GBM cells and healthy human astrocytes in the absence of US; this assessment emphasizes the safety of the plain nanoparticles, independently of their therapeutic activation, which in future applications can be performed with precise spatial focusing of US, ensuring that electric cues are generated only within the tumor tissue. Compared to conventional methods using broad electric fields, this dual control can provide a localized and safe modulation of cellular activity.^[^ ^53^ ^]^

Although ChNPs did not show any harmful effect even at the highest tested concentration (600 μg mL^−1^), a safety dose of 300 μg mL^−1^ was selected for all subsequent cell experiments. This choice was motivated by the observation that at 600 μg mL^−1^ visible nanoparticle aggregation occurred in the culture medium, potentially affecting cellular uptake and experimental consistency.

**Figure 3:**
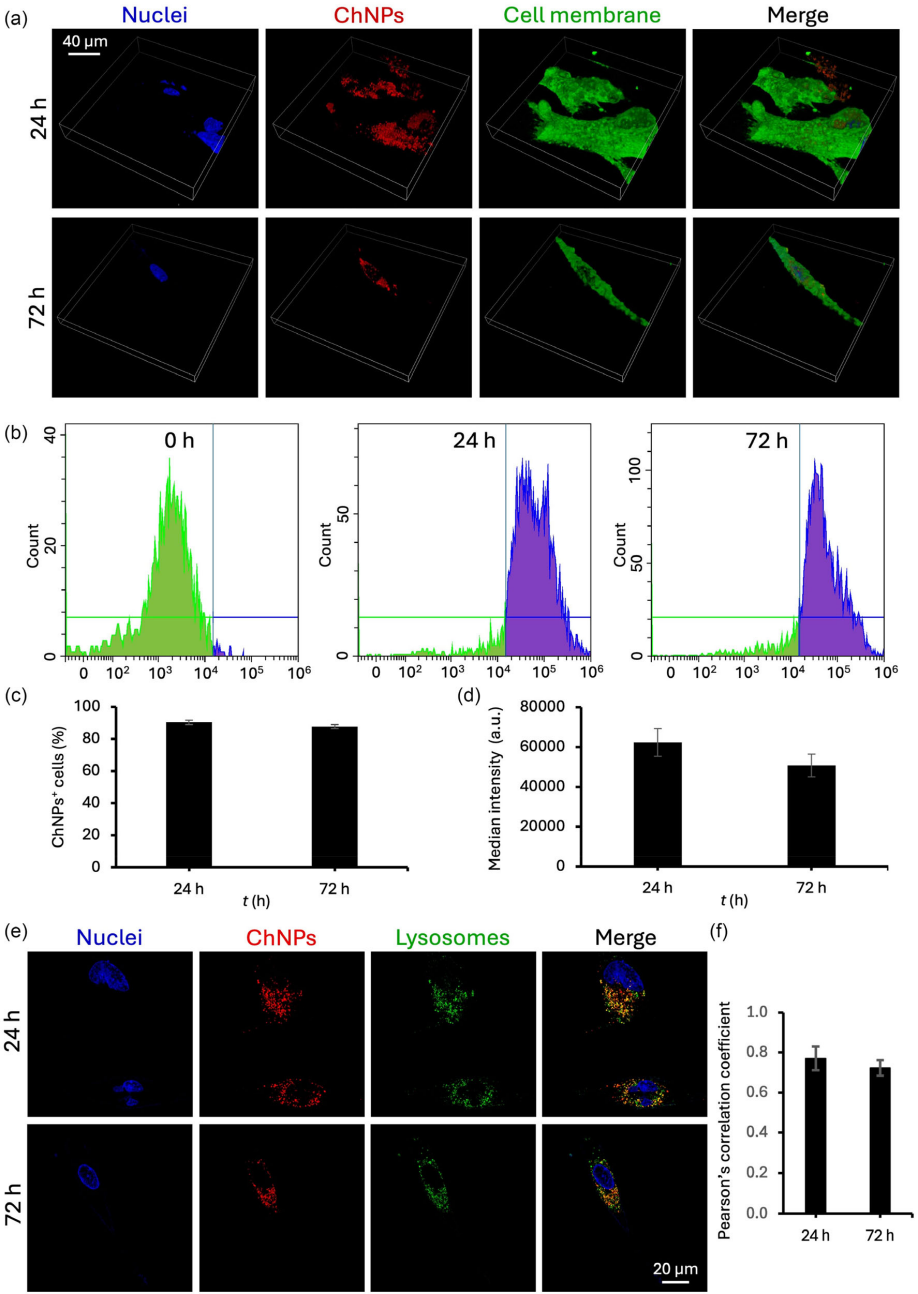
Nanoparticle internalization studies. a) Representative confocal microscopy Z‐stack images of GBM patient‐derived cells acquired at 24 and 72 h of incubation with ChNPs. b) Flow cytometry graphs suggesting the cellular internalization of ChNPs. Flow cytometry analysis of c) ChNPs^+^ cells and d) median fluorescence intensity. e) Representative confocal microscopy images at 24 and 72 h of incubation with ChNPs to assess lysosomal colocalization. f) Pearson's correlation coefficient analysis.

**Figure** **3** presents the analysis of the internalization and intracellular fate of ChNPs into patient‐derived GBM cells. In the representative confocal laser scanning microscopy (CLSM) images of Figure 3a, ChNPs are shown in red, the cell membranes in green, and nuclei in blue. At both 24 and 72 h of treatment, high levels of ChNPs were localized within the boundaries of the cell membrane, also appearing in close contact with the intracellular side of the membrane, suggesting efficient cellular association and internal retention. Flow cytometry analyses (Figure 3b) revealed a remarkable shift of the fluorescence peak at 24 h and maintained at 72 h compared to the 0 h control, confirming successful internalization. Quantitative flow cytometry analyses (Figure 3c) showed that over 85% of the cells remained ChNP‐positive at both time points (90 ± 1% at 24 h; 88 ± 7% at 72 h), while the median fluorescence intensity (Figure 3d) slightly decreased at 72 h (62000 ± 7000 arbitrary fluorescent units ‐AFU‐ at 24 h; 51000 ± 6000 AFU at 72 h), possibly due to lysosomal degradation and cell division. Representative CLSM images in Figure 3e demonstrate a strong signal overlap of ChNPs with acidic compartments (i.e., late endosomes and lysosomes) at both 24 and 72 h. The Pearson's correlation coefficient (Figure 3f) indicates high levels of colocalization (*r* > 0.7), albeit with a slight decrease at 72 h. These results indicate that ChNPs are effectively internalized by GBM cells and then partially trafficked into their acidic compartments.

### 2.3 Acute US‐Driven ChNPs‐Assisted Piezoelectric Stimulation

**Figure 4:**
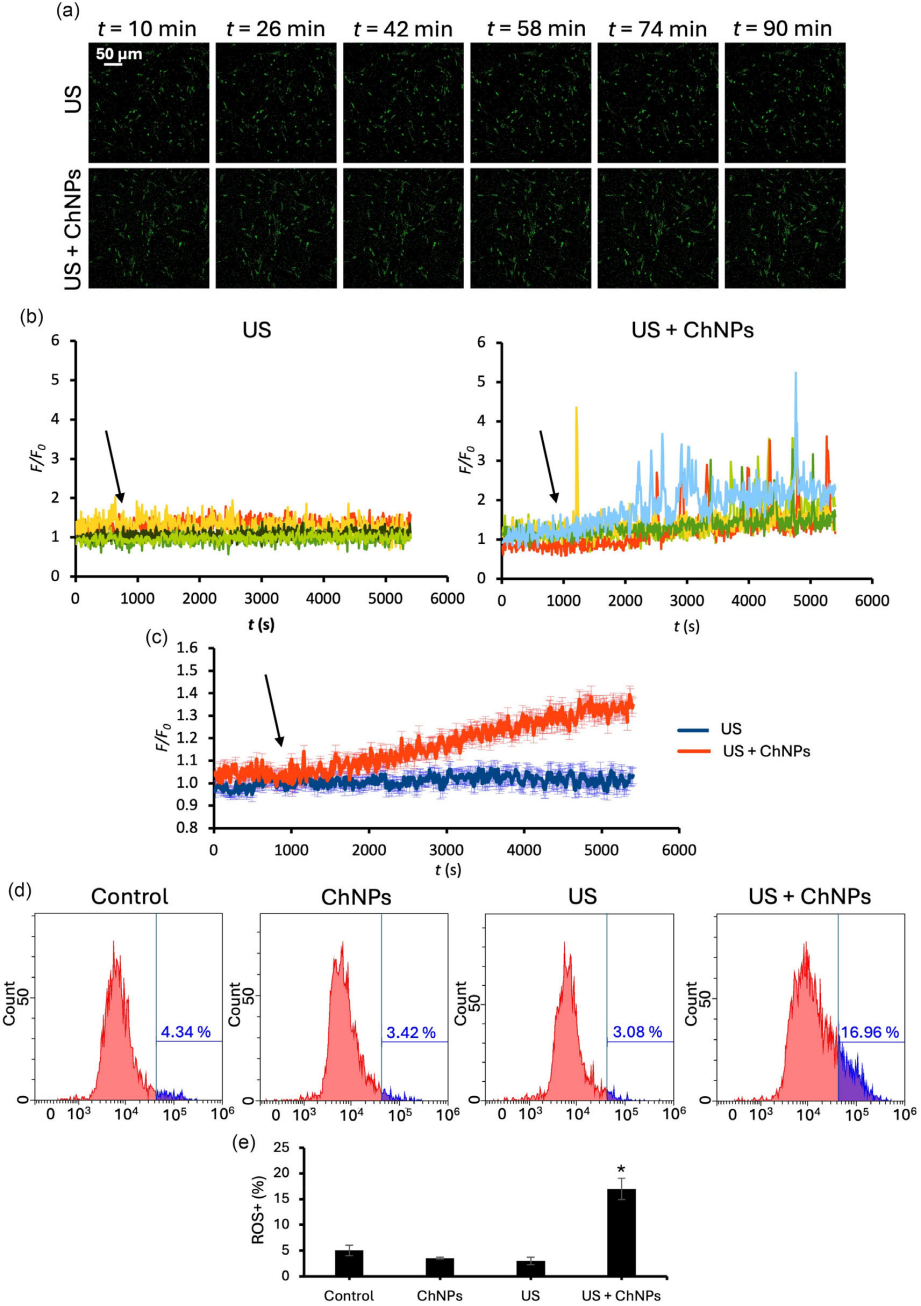
GBM cell activation in response to a single 1 h piezoelectric stimulation. a) Representative time‐lapse frames of the calcium imaging during US stimulation, in presence or absence of ChNPs. b) Representative *F/F*_0_ traces relative to Ca^2+^ imaging on patient‐derived GBM cells stimulated by US (left) and US + ChNPs (right). c) Average *F/F*_0_ traces relative to Ca^2+^ imaging on patient‐derived GBM cells stimulated by US (blue) and US + ChNPs (red). d) Representative distributions of ROS levels and e) % of ROS^*+*^ cells in patient‐derived GBM cells incubated with nanoparticles (ChNPs), stimulated with US (US), and exposed to combined treatment (US + ChNPs) compared to control cells (Control; * *p* < 0.001).

The effects of a single 1 h piezoelectric stimulation (US + ChNPs) on Ca^2+^ homeostasis and oxidative stress in GBM cells are presented in **Figure** **4**. Ca^2+^ imaging revealed minimal changes in intracellular Ca^2+^ levels upon US stimulation alone over the course of the experiment (Figure 4a, upper row), with *F/F*_0_ values remaining stable across time points, as also confirmed by the flat traces in individual and averaged fluorescence signals (Figure 4b,c, US condition). Conversely, GBM cells exposed to US upon incubation with ChNPs (Figure 4a, bottom row) exhibited a gradual and progressive increase in intracellular Ca^2+^, particularly visible after prolonged exposure (>40 min), indicating effective cell activation upon piezoelectric stimulation. Individual fluorescence traces (Figure 4b, right panel) showed the activation of multiple Ca^2+^ transients in the US + ChNPs condition, while averaged traces (Figure 4c) confirmed a significant increase in *F/F*_0_ compared to US alone starting after 900 s, when the stimulation has been initiated (black arrow), indicating the rise of the intracellular Ca^2+^ levels upon piezoelectric stimulation.

Normally, intracellular Ca^2+^ in nonstimulated GBM cells is tightly regulated, reflecting a balance between Ca^2+^ influx, efflux, and sequestration—processes primarily governed by voltage‐gated Ca^2+^ channels, plasma membrane Ca^2+^ ATPases, and endoplasmic reticulum Ca^2+^ pumps (SERCAs).^[^ ^54^, ^55^, ^56^, ^57^ ^]^ In the US + ChNPs condition, the slow but consistent increase in Ca^2+^ signal is incompatible with typical transient responses, and instead suggests sustained dysregulation. This result is consistent with the piezoelectric activation of cancer cells, as previously observed in breast cancer and GBM cells, and is an indicator of exit from cell cycle.^[^ ^20^, ^54^, ^55^ ^]^ Prolonged or aberrant Ca^2+^ increments are often interpreted by the cell as a stress signal, triggering pathways related to cell cycle arrest, autophagy, or even apoptosis.^[^ ^54^, ^55^ ^]^ Importantly, this sustained increase occurs without the use of pharmacological agents or high‐intensity stimuli, underscoring the efficacy of piezoelectric nanomaterials in modulating cellular function through remote, biocompatible stimulation. This mode of action is particularly relevant in the context of GBM, where the tumor microenvironment is characterized by stiffness, hypoxia, and resistance to conventional signals.^[^ ^2^, ^3^, ^6^, ^58^ ^]^ The capacity of US + ChNPs to perturb Ca^2+^ homeostasis suggests a possible strategy to sensitize resistant cancer cell phenotypes by disrupting calcium homeostasis, enhancing susceptibility to subsequent therapies.

Oxidative stress levels were evaluated by quantifying intracellular ROS upon single 1 h exposure to US, with or without preincubation with ChNPs. Flow cytometry analysis (Figure 4d,e) showed a significant increase in terms of ROS^+^ cells in the US + ChNPs group (≈17%, *p* < 0.001) compared to Control (≈4%), ChNPs alone (≈3%), and US alone (≈3%), supporting the hypothesis of the US‐driven ChNPs‐assisted piezocatalytic mechanism and in line with the results of Figure 1i.

Collectively, these findings demonstrate that ChNPs‐assisted piezoelectric stimulation induces both intracellular Ca^2+^ dysregulation (in terms of transients and basal levels) and ROS generation. The dual activation of Ca^2+^ and oxidative stress pathways could contribute to enhanced therapeutic efficacy.^[^ ^19^, ^21^, ^49^ ^]^ Importantly, neither ChNPs nor US alone significantly altered Ca^2+^ or ROS levels, confirming the necessity of their combination to achieve bioactive piezoelectric effects under the applied conditions. This highlights the safe and targeted nature of the piezostimulation, as biological activation occurs exclusively in the presence of both components (US + ChNPs).

### 2.4 Chronic US‐Driven ChNPs‐Assisted Piezoelectric Stimulation

**Figure 5:**
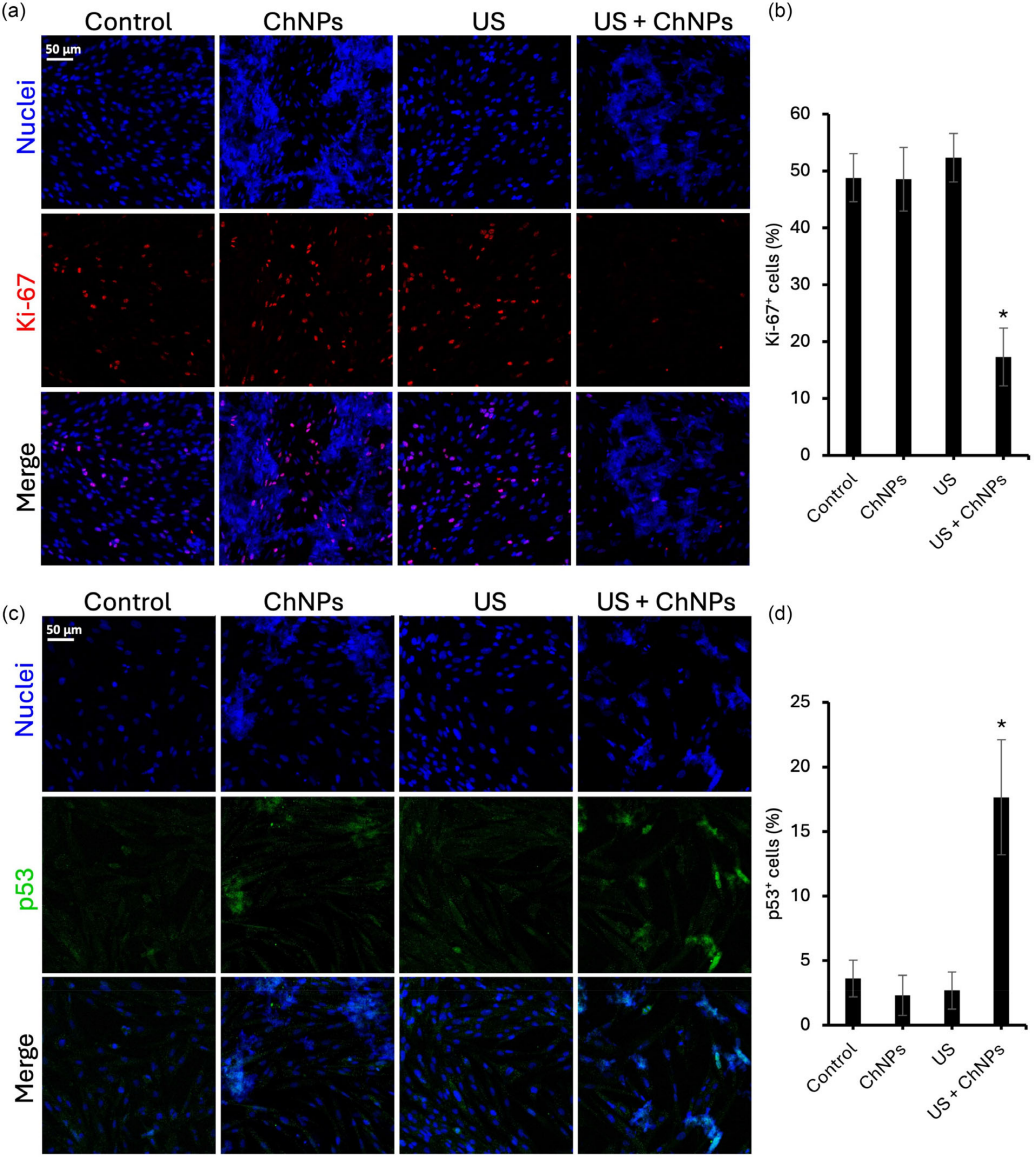
Cell proliferation and apoptosis analysis. a) Representative confocal images showing nuclear staining (blue) and Ki‐67 expression (red) in the four experimental conditions. b) Quantification of Ki‐67^+^ cells. c) Representative confocal images showing nuclear staining (blue) and p53 expression (green). d) Quantification of p53^+^ cells. *p < 0.001.

Chronic effects of a 3 day piezoelectric stimulation in patient‐derived GBM cells are reported in **Figure** **5**: specifically, the Ki‐67 proliferation‐associated marker^[^ ^59^, ^60^ ^]^ and the p53 apoptotic marker^[^ ^61^, ^62^, ^63^, ^64^, ^65^ ^]^ have been investigated.

As shown in Figure 5a,b, chronic piezoelectric stimulation with US + ChNPs resulted in a marked reduction in Ki‐67 expression compared to the Control group (*p* < 0.001). While the Control, ChNPs, and US treatments all maintained similar high levels of Ki‐67^+^ cells (49 ± 4%, 49 ± 6%, and 52 ± 4%, respectively), the combined US + ChNPs condition led to a pronounced decrease, with only 17 ± 5% of cells expressing Ki‐67.

Interestingly, the analysis of p53 expression (Figure 5c,d) revealed a biologically significant response unique to the US + ChNPs treatment. While cells exposed to Control, ChNPs, or US alone displayed consistently low levels of p53^+^ cells (4 ± 1%, 2 ± 2%, and 3 ± 1%, respectively), the group subjected to US stimulation in the presence of ChNPs showed a significant upregulation of p53 apoptotic marker, reaching 17 ± 5% of p53^+^ cells (*p* < 0.001). This result indicates a robust activation of cellular stress pathways, potentially linked to apoptosis or senescence.

Although previous studies have demonstrated p53 activation through piezoelectric stimulation, these effects were primarily achieved using inorganic nanoparticles that were either structurally modified or chemically functionalized to enhance their piezoelectric response,^[^ ^66^, ^67^ ^]^ or that required coadministration of chemotherapeutic agents to achieve significant biological effects.^[^ ^19^ ^]^ For instance, in^[^ ^66^ ^]^ BaTiO_3_ nanoparticles were poled to improve charge transfer, while in^[^ ^67^ ^]^ piezoelectric catalysis was mediated by BaTiO_3_ doped with iron ions to amplify ROS production. Our findings show for the first time that unmodified, fully organic and biodegradable chitosan‐based piezoelectric nanoparticles can elicit a comparable biological response, including p53 activation. This represents a significant step forward for the field of bioelectronic oncology, as it highlights the feasibility of using intrinsically biocompatible and clinically translatable materials to achieve therapeutic bioactivation via piezoelectric stimulation, without the need for inorganic or chemically engineered components.

To further investigate whether the observed p53 activation corresponds to apoptotic or senescent responses, annexin V‐FITC/propidium iodide (PI) flow cytometry analysis was performed on patient‐derived GBM spheroids after acute US stimulation (1 h). As shown in Figure S6, Supporting Information, the proportion of viable cells significantly decreased in the US + ChNPs group (77.6 ± 3.4%) compared to control (89.9 ± 0.4%; *p* < 0.05), while early apoptotic cells increased from 8.0 ± 0.7% to 19.5 ± 3.4% (*p* < 0.05). No significant change was observed in terms of late apoptotic or necrotic cells. These data confirm that piezoelectric stimulation primarily promotes apoptosis, in agreement with the upregulation of p53 observed at the protein level, thus supporting a proapoptotic rather than senescence‐related response.

**Figure 6:**
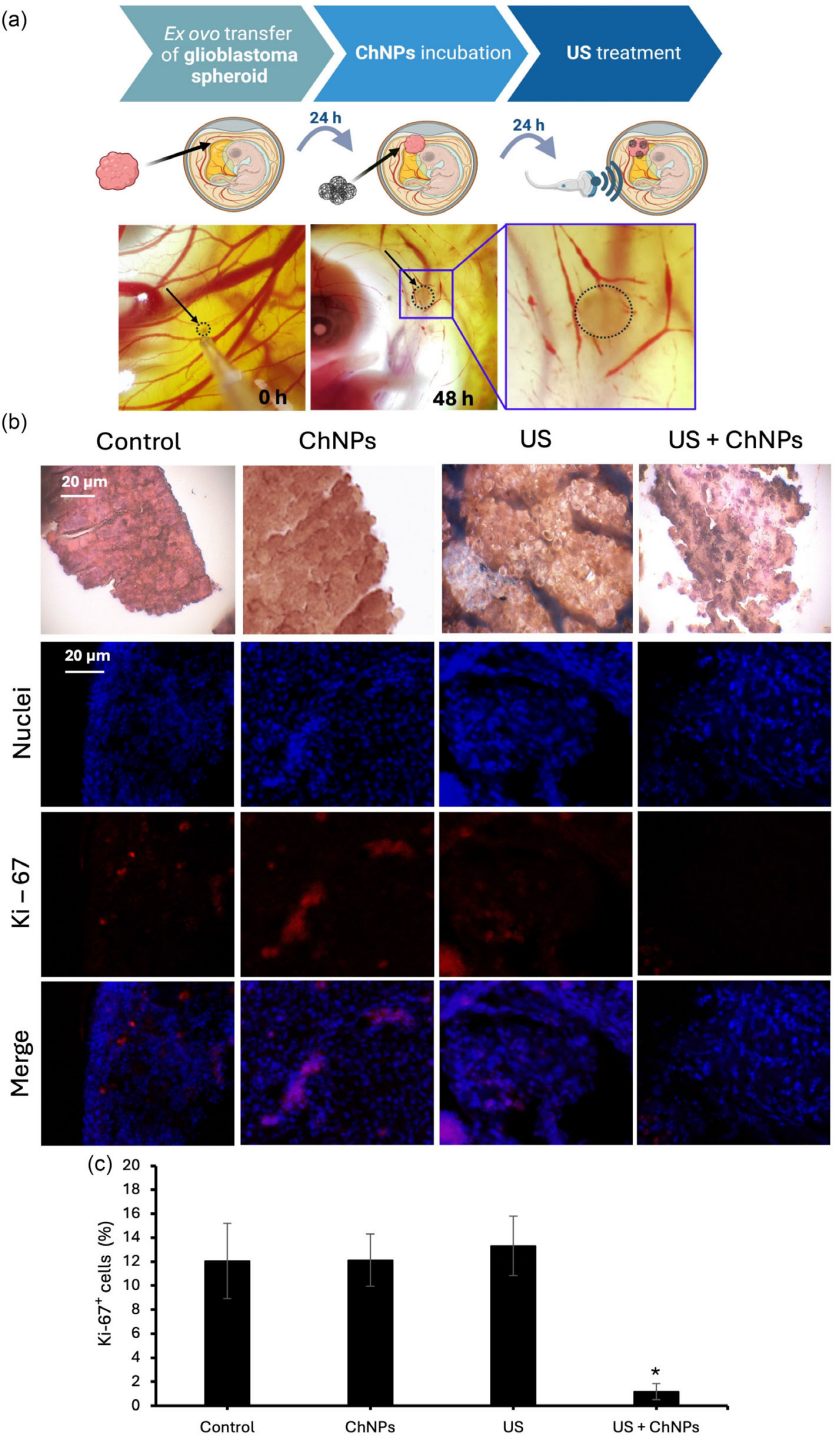
Ex ovo evaluation of GBM spheroid response to piezoelectric ChNPs and US treatment. a) Schematic representation of the experimental timeline: GBM spheroids were implanted onto the CAM of fertilized quail eggs, followed by incubation with ChNPs for 24 h and subsequent US treatment for 1 h; representative images of the CAM at 0 and 48 h show tumor localization (dotted circle). b) Histological (H&E staining; top row) and immunofluorescence analysis (Ki‐67 marker) of tumors retrieved from CAMs for different experimental conditions. c) Quantification of Ki‐67^+^ cells (%) for each experimental group (*p < 0.001).

**Figure** **6** reports the results of in vivo testing of piezoelectric ChNPs using the quail embryo chorioallantoic membrane (CAM) model, a biologically relevant and ethically accessible system for tumor nanomedicine studies.^[^ ^68^, ^69^, ^70^ ^]^ This platform is particularly suitable for hosting human cells and human cell‐derived spheroids due to its immunodeficient status at early developmental stages, allowing engraftment without rejection.^[^ ^70^ ^]^ The CAM model enabled dynamic monitoring of tumor treatment response within a physiologically vascularized context. Figure 6a reports the schematic overview of the experimental timeline: GBM spheroids were grafted into the CAM of fertilized quail eggs, followed by a 24 h incubation with ChNPs and a subsequent 1 h US stimulation.

Histological analysis (Figure 6b, top panels) reveals that tumor spheroids treated with the combined US + ChNPs exhibit a marked loss of tissue integrity compared to the other groups (Control, US, or ChNPs), which largely preserve their structural architecture. Immunofluorescence staining of the Ki‐67 marker showed a robust signal in Control (12 ± 3% of Ki‐67^+^ cells), ChNPs (12 ± 2% of Ki‐67^+^ cells), and US (13 ± 2% of Ki‐67^+^ cells) groups. Conversely, the US + ChNPs group exhibited a dramatic reduction in proliferative cells, with nearly complete loss of Ki‐67 expression (1 ± 1% of Ki‐67^+^ cells; Figure 6b, lower panels, and Figure 6c).

The significant reduction of Ki‐67 expression observed in the 3D CAM model following the combined US + ChNPs treatment demonstrates that the piezoelectric stimulation strategy is effective in a more physiologically relevant and dynamic setting. Unlike conventional 3D in vitro models, the CAM assay incorporates vascularization, extracellular matrix components, and host tissue interactions, offering a closer approximation to the in vivo tumor microenvironment. Overall, Ki‐67 expression levels in the 3D CAM model were lower than those observed in 2D in vitro cultures. This reduced proliferative index is consistent with the more physiologically constrained environment of the CAM, where gradients of oxygen, nutrients, and host‐derived signals contribute to mimicking vivo‐like tumor behavior more effectively than standard 3D culture systems.^[^ ^71^ ^]^

## 3 Conclusions

We have reported on the successful synthesis and characterization of the first known piezoelectric chitosan‐based nanoparticles, marking a significant advancement in the field of “smart” nanomaterials. Unlike literature examples based on inorganic or organic nonbiodegradable nanoparticles, ChNPs represent organic, biocompatible, biodegradable, and clinically approved materials, offering a safer and more sustainable approach for therapeutic development. The integration of piezoelectric properties into a biodegradable chitosan matrix paves the way for remote, noninvasive stimulation strategies that can be finely tuned through US modulation. Experiments using patient‐derived GBM cells revealed a strong therapeutic potential of ChNPs when combined with chronic US stimulation. Specifically, we observed a significant antiproliferative effect as indicated by the downregulation of Ki‐67, along with a notable proapoptotic response characterized by a consistent and robust upregulation of p53 expression. Importantly, these biological effects were achieved without the need for chemical cytotoxic agents, underscoring the promise of this physical‐stimulation‐based strategy in reducing off‐target toxicity and improving treatment specificity. The core novelty of this study lies in the intrinsic piezoelectric properties of chitosan‐based nanoparticles, directly conferred by a specific fabrication method, and by their exploitation in GBM therapy. These aspects represent a significant advancement in the field of nanomedicine with high translation potential, especially by taking into consideration that chitosan is already approved by regulatory agencies for several biomedical devices.

As a future perspective, our findings pave the way for preclinical testing in mammalian models and the development of wireless, noninvasive therapeutic platforms exploiting the unique properties of piezoelectric ChNPs. The potential to remotely activate antitumor responses via US presents a paradigm shift in precision oncology, particularly for tumors such as GBM, where surgical access and systemic treatment remain challenging. Additionally, the biodegradable nature of the ChNPs offers a substantial advantage in terms of safety, clearance, and clinical translation. Furthermore, given the intrinsic heterogeneity of patient‐derived GBM cultures, future studies may explore how different culture conditions, such as serum‐free versus serum‐containing media, affect the distribution of GBM cell subpopulations and their response to piezoelectric stimulation.^[^ ^72^ ^]^ Distinct glioma cell subtypes, including stem‐like and more differentiated phenotypes, may exhibit variable sensitivity to ChNP‐mediated activation: understanding these relationships will provide valuable insight into the robustness and selectivity of this therapeutic approach across the heterogeneous landscape of GBM.

Overall, this work not only introduces a novel class of bioactive nanomaterials but also opens new avenues for the development of next‐generation, stimuli‐responsive therapies tailored for aggressive cancers.

## 4 Experimental Section

4.1

4.1.1

##### Nanoparticle Fabrication

ChNPs were synthesized via an emulsion‐assisted alkaline precipitation method to obtain piezoelectric nanoparticles, as described in our patent.^[^ ^43^ ^]^ The used chitosan (Sigma‐Aldrich, 448869) shows a high degree of deacetylation (75%–85%, as described by the supplier), providing an adequate balance of solubility and crystallinity. While the degree of deacetylation can influence chain packing and hydrogen bonding, and thereby potentially affect crystallization and piezoelectric properties, the range used in this study is consistent with chitosan commonly used in biomedical research.^[^ ^73^, ^74^ ^]^ Chitosan was dissolved 1% w/v in an acidic aqueous solution of 5% v/v acetic acid. The solution was vortexed for 10 min to ensure complete polymer dissolution, then left to stand for 20 min to eliminate air bubbles. The oil phase was prepared by mixing dodecane (Sigma‐Aldrich) and Tween 85 (Sigma‐Aldrich) in a 10:1 ratio (total volume 25 mL). To initiate nanoparticle formation, 5 mL of the chitosan solution was added dropwise to the oil phase under constant homogenization at 15 000 rpm using a T10 basic ULTRA‐TURRAX homogenizer. After the complete addition of the aqueous phase, homogenization continued for an additional 2 min. Without stopping homogenization, 5 mL of a 50% w/v KOH solution (prepared by dissolving 50 g of KOH in 50 mL of distilled water) was added dropwise using a glass pipette. This addition induced phase separation, enabling recovery of the aqueous phase containing the nanoparticles. The oil phase was removed using a glass pipette, and the aqueous phase was transferred to a 50 mL plastic centrifuge tube and brought to volume with Milli‐Q water. Nanoparticles were purified by centrifugation at 11200 g for 15 min using a Hettich Universal 320/320 R centrifuge. The resulting pellet was resuspended in fresh Milli‐Q water, and the washing step was repeated six times to ensure the removal of residual contaminants. Finally, the purified ChNPs were resuspended in 5 mL of Milli‐Q water and sonicated using a Fisherbrand Q125 Sonicator at 35% amplitude for 40 min. Sonication was performed with the vial immersed in an ice‐water bath to maintain temperature and prevent overheating. Figure S1, Supporting Information, shows a schematic of the ChNPs fabrication.

ChNPs* were obtained by ionotropic gelation. A 1% w/v chitosan solution was prepared by dissolving chitosan (Sigma‐Aldrich) in 5 mL of a 5% v/v citric acid aqueous solution under magnetic stirring (300 rpm) until complete dissolution. Tween 85 (Sigma‐Aldrich) was then added to a final concentration of 0.3% w/v and mixed until fully solubilized. In parallel, a 0.1% w/v tripolyphosphate (TPP) (Fisher Scientific) solution was prepared in 5 mL of milliQ water, and its pH was adjusted to 5.0 using HCl or NaOH. The TPP solution was added dropwise to the chitosan solution under magnetic stirring at room temperature, allowing spontaneous nanoparticle formation via electrostatic interactions between the protonated amino groups of chitosan and the negatively charged phosphate groups of TPP. Nanoparticles were stirred for 30‐60 min to promote stabilization. The suspension was then sonicated using a Fisherbrand Q125 Sonicator at 35% amplitude for 40 min. Finally, ChNPs* were purified by five consecutive centrifugation steps at 11200 g for 10 min each using a Hettich Universal 320/320 R centrifuge, with thorough resuspension in milliQ water after each cycle.

##### Nanoparticle Characterization

SEM analysis was performed on a Helios NanoLab 600i FIB/SEM, FEI. A 5 μL drop of the samples, prepared at a concentration of 5 μg mL^−1^, was deposited onto a silicon wafer and left to air dry. The dried samples were then coated with a thin layer of gold using a Quorum Tech Q150RES Gold Sputter Coater, set to 10 mA for 30 s. Images were subsequently acquired.

Hydrodynamic size, PDI, and ζ‐potential measurements were obtained using a Zeta‐sizer NanoZS90 (Malvern Instruments Ltd.). Dispersions were prepared at a concentration of 100 μg mL^−1^ in ultrapure MilliQ water; hydrodynamic diameter and PDI measurements were performed using disposable polystyrene cuvettes, while ζ‐potential measurements were conducted in folded capillary cells (Malvern Zetasizer Nano series). Each measurement was carried out three times, and the mean and standard deviation were computed based on these replicates.

Concerning XRD analysis, a volume of about 0.25 cm^3^ of a 300 μg mL^−1^ ChNP water dispersion was freeze‐dried (Freezone 2.5 liter −84C Benchtop Freeze dryer, Labconco), and the resulting dry powder was gently mounted onto a zero‐background silicon sample holder for XRD analysis. XRD patterns were acquired using a Malvern‐PANalytical Empyrean 3rd generation X‐ray powder diffractometer, equipped with a 1.8 kW CuKα radiation source (*λ* = 1.5406 Å) operating at 45 kV and 40 mA. The system featured iCore and dCore PreFIX optical modules and a PIXcel3D solid‐state hybrid pixel area detector operating in 0D mode. Data were collected over a *2θ* range of 5° to 40°, with a step size of 0.0525°, using a parallel beam geometry. The configuration included 0.04 rad Soller slits and a 0.28° parallel plate collimator on the diffracted beam, optimized for samples with highly irregular surfaces. All measurements were performed in ambient air at room temperature.

The piezoelectric properties were investigated using PFM. Droplets of the diluted samples (50 μg mL^−1^ in Milli‐Q water) were placed onto a conductive silicon wafer and allowed to dry. Measurements were conducted using an atomic force microscope equipped with a conductive tip specific for PFM (ASYELEC.01‐R2, Oxford Instruments), operating in contact mode for both PFM and AFM. PFM measurement was performed off‐contact resonance, with parameters including an applied voltage of 1 V, a scan area of 5 μm, a scan rate of 0.6 Hz, a tip oscillation frequency of 75 kHz, and a scan speed of 7.15 μm s^−1^. AFM parameters were the same used for PFM, but no voltage was applied across the samples. The piezoelectric coefficient *d*_33_ was derived by characterizing the individual particles in PFM mode and calculating the deformation value at the center of the single particles; the measurement was repeated for 15 ChNPs and then the average *d*_33_ was calculated.

To evaluate the degradation of ChNPs, two experimental groups were prepared: PBS (pH 7.4) and a solution adjusted to pH 5.8, both with a nanoparticle concentration of 300 μg mL^−1^. The acidic solution was prepared by adjusting PBS pH to 5.8 with NaH_2_PO_4_ and adding H_2_O_2_ to a final concentration of 200 μM, to mimic lysosomal conditions. Samples were kept at 37 °C and collected at different time points and analyzed by SEM upon dilution (1:1000) and UV–vis spectroscopy (Perkin Elmer Victor X3 UV–vis spectrophotometer). The total number of nanoparticles measured for size analysis was 1310; control = 302; 24 h at pH 5.8 = 170; 72 h at pH 5.8 = 252; 144 h at pH 5.8 = 111; 24 h at pH 7.4 = 35; 72 h at pH 7.4 = 228; 144 h at pH 7.4 = 212. Figure S7a, Supporting Information, shows the UV–vis absorption spectrum of ChNPs at a concentration of 500 μg mL^−1^, recorded in the wavelength range of 400–700 nm (measurements were performed in PBS buffer at pH 7.4); a calibration curve was generated by correlating ChNP concentration with absorbance at 400 nm (Figure S7b, Supporting Information), enabling the quantification of nanoparticle concentration over time based on absorbance measurements.

To evaluate the piezocatalytic effect of ChNPs, 2 mL of dispersion (concentration of 300 μg mL^−1^) was incubated with a singlet oxygen probe (Singlet Oxygen Sensor Green, Invitrogen). Experiments were conducted following the manufacturer's instructions (Invitrogen) for Singlet Oxygen Sensor Green, with excitation/emission settings comparable to fluorescein. The samples were exposed to US treatment for 2 h at room temperature. US stimulation was applied using a KTAC‐4000 device (Sonidel) connected to a planar US transducer (20 mm diameter), set to a power density of 1 W cm^−2^, frequency of 1 MHz, burst rate of 0.5 Hz, and duty cycle of 10%. Fluorescence intensity was measured using a Victor3 microplate reader (PerkinElmer) at *λ*_ex_ = 485 nm and *λ*_em_ = 535 nm to assess singlet oxygen generation. Moreover, emission spectra of the representative samples were collected with a fluorescence spectrophotometer (Agilent Technologies; *λ*_ex_ = 485, *λ*_em_ = 495‐800 nm). For each experimental group, measurements were performed in triplicate. The blank, consisting of PBS and the singlet oxygen sensor, was subtracted from each sample. Data were reported as percentage change relative to the control. To assess piezocatalytic behavior under reductive conditions, reduced L‐glutathione (GSH; Merck) was added to the ChNP dispersion or to PBS at a final concentration of 10 mM, consistent with reported concentrations in the tumor microenvironment.^[^ ^52^ ^]^ Samples were then subjected to the same US protocol described above, and fluorescence measurements were acquired under identical conditions.

##### Cell Derivation and Culture

Primary GBM cell cultures were obtained from resected tissue samples from patients diagnosed with grade IV primary glioma, IDH‐1 wild type, at San Martino Hospital (Genova, Italy), following informed consent and local ethical guidelines (Liguria Ethics Committee Registration CER 341/2019), as previously described.^[^ ^75^ ^]^ Briefly, immediately postsurgery, the tissue samples were transferred into 15 mL tubes containing sterile saline solution and kept at 4 °C until processing. Upon arrival at the lab, samples were washed twice with Dulbecco's phosphate buffered saline (DPBS) (Euroclone) and mechanically dissociated. Tissue fragments were placed in a 10 cm Petri dish (Corning) containing 5 mL of DPBS, then finely chopped with a scalpel. The cell suspension was transferred into a 15 mL tube containing 3 mL of collagenase (Collagenase Type I, Sigma‐Aldrich) and incubated under continuous agitation at 37 °C for 10–12 min. Following enzymatic digestion, the suspension was centrifuged at 600 g for 5 min, and the supernatant was discarded. The resulting pellet was resuspended in 2 mL of 0.05% trypsin‐EDTA solution (Gibco) and incubated for 3 min at 37 °C. Cells were then centrifuged again at 600 g for 5 min, and the supernatant was removed. The pellet was subsequently resuspended in 5 mL of complete medium and filtered through a 70 μm cell strainer into a 50 mL conical tube to remove cell aggregates and undigested tissue fragments. The filtered suspension was then transferred into a clean 15 mL conical tube and centrifuged at 600 g for 5 min. After discarding the supernatant, 800 μL of 10× Red Blood Cell lysis buffer (Abcam) was added to the pellet, and the suspension was gently pipetted for 20–30 s to ensure homogeneous mixing. The sample was centrifuged once more at 600 g for 5 min. Finally, the supernatant was removed, and the resulting cell pellet was plated in a T25 cm^2^ flask and then cultured with 10 mL of high‐glucose Dulbecco's modified Eagle medium/F‐12 (DMEM/F‐12, Sigma‐Aldrich) supplemented with penicillin/streptomycin (100 IU mL^−1^ penicillin and 100 μg mL^−1^ streptomycin, Gibco), 1% L‐glutamine (Gibco), and 10% fetal bovine serum (FBS) (Sigma‐Aldrich). This mixture is hereafter referred to as “complete medium.” The complete medium supplemented with serum is able to sustain heterogeneity of patient‐derived cell subpopulations, including *β*‐III‐tubulin^+^ and GFAP^+^ cells, as well as nestin^+^ and vimentin^+^ cancer stem‐like cells (although these are less represented than in serum‐free conditions), reflecting the intra‐ and intertumoral variability.^[^ ^72^ ^]^

After three days, patient‐derived GBM cultures were washed to remove debris and refreshed with complete medium. When cultures reached ≈80% confluence, cells were washed with DPBS without calcium and magnesium (Euroclone), treated with 2 mL of 0.05% trypsin/EDTA (Gibco) for 7 min, centrifuged at 610 g for 5 min, and reseeded in 10 cm Petri dishes containing 10 mL of complete medium. Cultures were maintained at 37 °C in a humidified atmosphere with 5% CO_2_. The culture medium was refreshed every two days, and cells were subcultured upon reaching 80%–90% confluence.

To generate GBM spheroids, a 96‐well plate was prepared by adding 75 μL of preheated 1% w/v agarose into each well; as the agarose solidifies, it forms a nonadherent meniscus. Meanwhile, patient‐derived GBM cells were counted using a Burker chamber, and seeded into the wells at densities of 10000 cells/well in a total volume of 150 μL per well. The culture medium was refreshed by replacing half of the volume every couple of days.

Immortalized human astrocytes, obtained from Innoprot, were cultured using the Human Astrocyte Growth Medium Kit (Cell Applications, INC), which included a basal medium and growth supplement, further supplemented with 1% penicillin/streptomycin solution (100 IU mL^−1^ penicillin and 100 μg mL^−1^ streptomycin, Gibco). Cultures were maintained under sterile conditions at 37 °C in a humidified atmosphere at 5% CO_2_. The culture medium was refreshed every 48 h, and cells were passaged using 0.05% trypsin‐EDTA solution (Gibco).

##### Nanoparticle Cytocompatibility

Cell viability was evaluated using the WST‐1 assay (2‐(4‐iodophenyl)‐3‐(4‐nitrophenyl)‐5‐ (2,4‐disulfophenyl)‐2H‐tetrazolium monosodium salt, in a premix electrocoupling solution, BioVision) to assess metabolic activity following ChNP incubation at varying concentrations and to determine the maximum nontoxic dose suitable for cell stimulation experiments. Biocompatibility studies were performed to evaluate the intrinsic safety of ChNPs without the application of US. This approach was chosen to assess potential off‐target effects of the nanomaterial on healthy cells, such as human astrocytes. Combined US + ChNP stimulation was not applied to healthy cells, as in clinical practice US can be precisely focused on the tumor area, minimizing exposure of surrounding healthy tissue (e.g., see localized brain cancer infusion and focused US techniques). Five concentrations were tested (0, 100, 200, 300, and 600 μg mL^−1^); a 96‐well plate was used, with six replicates per concentration. The day following seeding, the medium was removed from each well and replaced with medium containing the corresponding concentration of ChNPs. At 72 h of incubation with the ChNPs, the WST‐1 assay was performed. Culture medium was removed, and each well was treated with 100 μL of a WST‐1 solution (diluted 1:11 in phenol red‐free DMEM with 10% FBS). The plate was incubated for 40 min, after which 80 μL from each well was transferred to a new 96‐well plate for absorbance reading. Absorbance was measured at 450 nm using a Victor3 microplate reader (PerkinElmer), and metabolic activity for each condition was normalized against the absorbance of the control cultures.

##### Nanoparticle Internalization

To assess ChNP internalization, nanoparticles were labeled with rhodamine B isothiocyanate (RITC) (Sigma‐Aldrich, 1.5 mg mL^−1^ in ethanol) at a 1:15 w/w dye‐to‐ChNPs ratio. RITC is commonly used to label chitosan because its isothiocyanate group selectively reacts with primary amines on the polymer backbone, forming stable thiourea bonds and enabling covalent fluorescence labeling.^[^ ^76^ ^]^ ChNPs (1.2 mg mL^−1^) were stirred in PBS at 300 rpm for 2 h at room temperature, then centrifuged to remove excess dye and resuspended in culture medium at 300 μg mL^−1^.

Patient‐derived GBM cells were seeded in 24‐well μ‐Plates (Ibidi) (3·10^4^ cells/cm^2^) in 500 μL of culture medium per well. All experiments were performed in triplicate for both the 24‐ and 72 h time points. At 24 h after seeding, cells were incubated with fluorescent ChNPs suspended in the culture medium at a concentration of 300 μg mL^−1^. At the different time points, cells were detached with 180 μL of 0.05% trypsin‐EDTA solution (Gibco) after washing in PBS without calcium and magnesium. Trypsin‐EDTA solution was diluted with 400 μL of culture medium for each well, and the content was collected and centrifuged for 7 min at 600 g. The cell pellet was resuspended in 300 μL of PBS and analyses performed using a flow cytometer (Beckman Coulter CytoFLEX) to assess the percentage of cells positive for fluorescence (*λ*_ex_ = 488 nm, *λ*_em_ = 585 ± 21 nm). The autofluorescence threshold was determined by control data (0 h exposure); data were processed using CytoFLEX software.

Analogous cultures were also exploited for confocal imaging; colocalization of ChNPs with lysosomes was assessed using LysoTracker Deep Red dye (Invitrogen) staining solution (0.5 μL of fluorescent dye was added to 500 μL of cell medium). Cells were incubated with the LysoTracker Deep Red dye for 50 min; then, cell nuclei were fluorescently labeled by adding 0.5 μL of Hoechst 33342 to the staining solution and incubating for a further 10 min. For the investigation of nanoparticles‐cell membrane interaction, cells were stained with CellMask Green Plasma Membrane Stain (1:1000 dilution, Invitrogen) and Hoechst 33342 (1:1000 dilution) in complete medium for 15 min. The fluorescent medium was then removed and replaced with 500 μL of phenol red‐free DMEM supplemented with 10% FBS. Confocal imaging was carried out using a CLSM system (C2s, Nikon).

##### Oxidative Stress Analysis

To evaluate ROS generation upon piezoelectric stimulation, patient‐derived GBM cells were seeded at a density of 3·10^4^ cells per well in 48‐well plates and divided into four experimental groups, with six replicates per group: Control (no stimulation, culture medium without nanoparticles), ChNPs (no stimulation, culture medium with nanoparticles 300 μg mL^−1^), US (US stimulation, culture medium without nanoparticles), and US + ChNPs (US stimulation, culture medium with nanoparticles 300 μg mL^−1^). The groups assigned to US treatment (US and US + ChNPs) were exposed to US for 1 h: US stimulation was applied as previously described^[^ ^19^, ^20^, ^21^, ^29^, ^44^ ^]^ using a KTAC‐4000 device (Sonidel) connected to a planar US transducer (20 mm diameter), set to a power density of 1 W cm^−2^, frequency of 1 MHz, burst rate of 0.5 Hz, and duty cycle of 10%. These settings were selected to avoid nonspecific mechanical and temperature stimulations to the cells.

The cultures were then rinsed with PBS, detached from the plates using 0.05% trypsin‐EDTA solution (Gibco), and centrifuged at 1000 g for 7 min at 4 °C. Thereafter, the cells were stained with 5 mM CellROX Green Reagent (Invitrogen) for 15 min in PBS; the fluorescence intensity was eventually measured using a Beckman Coulter CytoFLEX (*λ*_ex_ = 488 nm; *λ*_em_ = 525 ± 20 nm).

##### Calcium Imaging

Patient‐derived GBM cells were seeded in 24‐well μ‐Plates (Ibidi) with a density of 3·10^4^ cells/cm^2^. Two experimental groups were established: one receiving US stimulation in standard culture medium (US), and the other receiving US in the presence of ChNPs (US + ChNPs). At 24 h postseeding, ChNPs were added to the US + ChNPs group at a final concentration of 300 μg mL^−1^, while the medium in the US group was refreshed to maintain consistency. After an additional 24 h, cells were stained with Fluo‐4 AM (1 μM, Invitrogen) for 40 min at 37 °C. Following incubation, cells were rinsed with PBS and placed in phenol red‐free DMEM with HEPES (25 mM, Thermo Fisher) for time‐lapse fluorescence imaging using a Nikon Eclipse Ti‐E epifluorescence microscope. Ca^2+^ flux were monitored 15 min before the onset of US stimulation and extending 15 min after its conclusion to capture cellular activity both with and without stimulation. Chronic US stimulation was applied as described in Section 3.6. Fluorescence intensities were calculated by averaging pixel values within the intracellular region of interest. Baseline fluorescence intensity, denoted as *F*_0_, was measured at time *t* = 0 s, while intensities at subsequent time points (*t* > 0 s) were represented as *F*. *F/F*_0_ ratios were plotted to illustrate fluorescence traces under both experimental conditions (US and US + ChNPs) throughout the time‐lapse experiment. Representative images from both experimental groups were selected at defined time points to show the Ca^2+^ flux over time.

##### Chronic Piezoelectric Stimulation

Chronic piezoelectric stimulation was performed using the same US protocol repeated for three consecutive days, with 1 h sessions per day. Patient‐derived GBM cells were seeded with a density of 4.8·10^4^ cells/cm^2^ in 24‐well μ‐Plates (Ibidi) and divided into four experimental groups: Control (no stimulation, culture medium without nanoparticles), ChNPs (no stimulation, culture medium with nanoparticles), US (US stimulation, culture medium without nanoparticles), and US + ChNPs (US stimulation, culture medium with nanoparticles). 24 h after seeding, cells in the ChNPs and US + ChNPs experimental classes were treated with ChNPs at a concentration of 300 μg mL^−1^, while the medium in the control and US experimental classes was just replaced. 48 h after seeding, US stimulation was applied to the US and US + ChNPs experimental classes for 1 h for three consecutive days. At the end of the experiment, cultures were fixed using 4% w/v paraformaldehyde at 4 °C for 20 min, preparing them for immunofluorescence analysis.

The expression of two markers, Ki‐67 (for proliferation) and p53 (for apoptosis), was assessed by immunofluorescence. Cell membranes were permeabilized with Triton X‐100 (0.1% in PBS) for 1 h at room temperature, and blocking was carried out with 10% goat serum in PBS for 1 h. The cultures were then incubated with primary antibodies: rabbit IgG anti‐Ki‐67 (diluted 1:150 in 10% goat serum for 1 h at 37 °C, Abcam) or mouse IgG anti‐p53 (diluted 1:150 in 10% goat serum for 2 h at 37 °C, Abcam). After incubation, the cells were washed twice with 10% goat serum in PBS and then stained with a solution containing Hoechst 33342 (1:500, Invitrogen), to counterstain cell nuclei, and a fluorescently labeled secondary antibody (anti‐mouse or anti‐rabbit, 1:250, Invitrogen), in 10% goat serum in PBS. Imaging was then carried out using a CLSM system (C2s, Nikon).

Flow cytometry analysis for the investigation of apoptotic phenomena on 3D spheroids was carried out similarly to previously described.^[^ ^77^ ^]^ Briefly, after trypsin treatment (10 min at 37 °C), the cell suspensions were treated with buffer containing 2.5 μM annexin V‐FITC and 1 μg mL^−1^ PI. Samples were incubated for 20 min at 37 °C in the dark and subsequently analyzed using a Beckman Coulter CytoFLEX cytometer (FITC channel: *λ*_ex_ = 488 nm, *λ*_em_ = 525 ± 40 nm; PI channel: *λ*_ex_ = 488 nm, *λ*_em_ = 690 ± 50 nm). The proportions of viable, early apoptotic, late apoptotic, and necrotic cells were determined using CytExpert software and expressed as mean ± standard deviation from triplicate measurements.

##### Ex ovo Model Experiments

Fertilized quail eggs (*Coturnix japonica*, Japocaille) were incubated in an automatic egg incubator at 37 °C with 57% humidity for 3 days. On the third day, the eggs were opened with pointed tweezers, and each embryo was transferred to a Petri dish and incubated at 37 °C for an additional 3 days. To graft the spheroids, a small incision was made in the CAM using a needle, and a spheroid was placed onto it with a pipette. The embryo was then returned to the incubator to allow the spheroids to vascularize. The same experimental groups described for in vitro testing were set up; one day after the spheroid grafting, ChNPs‐treated samples were administered with 10 μL of ChNP dispersion in complete medium (300 μg mL^−1^), added with a pipette just over the spheroid. For the experimental classes stimulated with US, after a further 24 h the Petri dish was filled with PBS and then irradiated with US for 1 h using a KTAC‐4000 device (Sonidel) connected to a planar US transducer (20 mm diameter), set to a power density of 1 W cm^−2^, frequency of 1 MHz, burst rate of 0.5 Hz, and duty cycle of 10%.

At the end of the experimental procedure, the grafted spheroids were extracted using a scalpel and a tweezer and fixed using 4% w/v paraformaldehyde at 4 °C for 24 h, rinsed 4 times with PBS for 20 min each, and then immersed in 70% ethanol. The embedding procedure was carried out in a 42 °C water bath, with all steps performed in 1.5 mL Eppendorf tubes placed in floating racks on a heated plate. Samples were dehydrated through a graded ethanol series, beginning with 80% ethanol for 10 min, followed by two changes in 95% ethanol for 25 min each. Dehydration was completed with three consecutive rinses in 100% ethanol, each lasting 30 min. Tissues were then cleared in xylene (Fisher Scientific) at 42 °C, with two rinses of 25 min. After complete clearing, xylene was fully removed, and the samples were infiltrated with paraffin overnight at 58–60 °C in metal molds. The following day, the molds were transferred to a room‐temperature plate for 3 h, then cooled at –20 °C for 30 min to allow solidification. Once solid, the paraffin blocks were carefully removed from the molds and prepared for sectioning. Sectioning was performed using a rotary microtome (Histo‐Line Laboratories, Libra, MR3000) equipped with a razor‐sharp blade. Microscope slides were precoated with a thin layer of distilled water and placed on a heating plate set to 40–45 °C. Sections were gently transferred onto the prewarmed slides and allowed to dry for 10 min; excess water was removed using a plastic pipette. Slides were finally baked overnight at 37 °C to ensure proper adhesion of the sections and remove the residual water. Paraffin removal was performed by immersing slides in two successive xylene baths for 10 min each. Three consecutive 3 min rinses in 100% ethanol were performed; finally, slides were rehydrated in distilled water and kept immersed until further immunostaining procedures.

The slides were permeabilized with Triton X‐100 (0.1% in PBS) for 1 h at room temperature, and blocking was done with 10% goat serum in PBS for 1 h. The sections were then incubated with the primary antibody (rabbit IgG anti‐Ki‐67, diluted 1:150 in 10% goat serum for 1 h at 37 °C, Abcam). After incubation, the slides were washed three times with 10% goat serum in PBS and then stained with a solution containing 10% goat serum in PBS, Hoechst 33342 (1:500, Invitrogen) to mark cell nuclei, and a fluorescently labeled secondary antibody (anti‐rabbit, 1:250, Invitrogen). Imaging was carried out using a CLSM system (C2s, Nikon).

For hematoxylin and eosin (H&E) staining, sections were immersed in hematoxylin for 5 min, rinsed in tap water for 10 min, then stained with eosin for 1 min and rinsed with Milli‐Q water. Subsequently, slides were dehydrated in 95% ethanol for 5 min. Images were acquired using a Hirox HRX‐01 3D digital optical microscope.

##### Statistical Analysis

All data were presented as mean ± standard deviation. Statistical analyses were conducted using either *R* software (version 4.0.3) or Microsoft Excel (Microsoft 365). Comparisons between two groups were performed in Excel using a two‐tailed Student's t‐test. For comparisons involving more than two groups, analyses were carried out in *R* using one‐way ANOVA followed by Fisher's least significant difference test with Bonferroni's *post‐hoc* correction. Statistical significance was set at *p* < 0.001.

## Supporting Information

Supporting Information is available from the Wiley Online Library or from the author.

## Conflict of Interest

Marino A., Curiale T., Ziaja K., Torre B., and Ciofani G. are inventors on the patent “Nanoparticelle piezoelettriche di chitosano e loro usi in applicazioni biomediche” (IT102025000004170, filed on 28/02/2025). The authors declare no other competing interests.

## Supporting information

Supplementary Material

## Data Availability

The data that support the findings of this study are available from the corresponding author upon reasonable request.
