# Supplementary material for: Ultrasound‐Activated Biodegradable Piezoelectric Chitosan Nanoparticles for Glioblastoma Treatment
Source: Small Sci. 2025 Nov 21;6(1):e202500457. doi: 10.1002/smsc.202500457 (PMC12798788; doi:10.1002/smsc.202500457)
Supplement: Supplementary file 1 — Supplementary Material [file SMSC-6-e202500457-s001.pdf]

SUPPORTING INFORMATION FOR

Ultrasound-Activated Biodegradable Piezoelectric Chitosan Nanoparticles for Glioblastoma Treatment

Attilio Marino[1,†,], Tommaso Curiale[1,†,*], Marie Celine Lefevre[1], Alessio Carmignani[1], Maria Cristina Ceccarelli[1,2], Matteo Battaglini[1], Kamil Ziaja[1,2,3], Sergio Marras[4], Bruno Torre[5], Pietro Fiaschi[6,7], Gianni Ciofani[1,*]*

[1] Istituto Italiano di Tecnologia, Smart Bio-Interfaces, Viale Rinaldo Piaggio 34, 56025 Pontedera, Italy
[2] Scuola Superiore Sant'Anna, The Biorobotics Institute, Viale Rinaldo Piaggio 34, 56025 Pontedera, Italy
[3] University of Aveiro, Department of Chemistry, CICECO-Aveiro Institute of Materials, Rua de Calouste Gulbenkian 1, 3810-074 Aveiro, Portugal
[4] Istituto Italiano di Tecnologia, Nanochemistry Department, Via Morego 30, 16130 Genova, Italy
[5] Istituto Nazionale Di Ricerca Metrologica, Strada delle Cacce 91, 10135 Torino, Italy
[6] IRCCS Ospedale Policlinico San Martino, Department of Neurosurgery, Largo Rossana Benzi 10, 16132, Genova, Italy
[7] University of Genova, Department of Neuroscience, Rehabilitation, Ophthalmology, Genetics, Maternal and Child Health (DiNOGMI), Largo Paolo Daneo 3, 16132, Genova, Italy

[†] Authors contributed equally to this work and shared first authorship
* Corresponding Authors: attilio.marino@iit.it; tommaso.curiale@iit.it; gianni.ciofani@iit.it

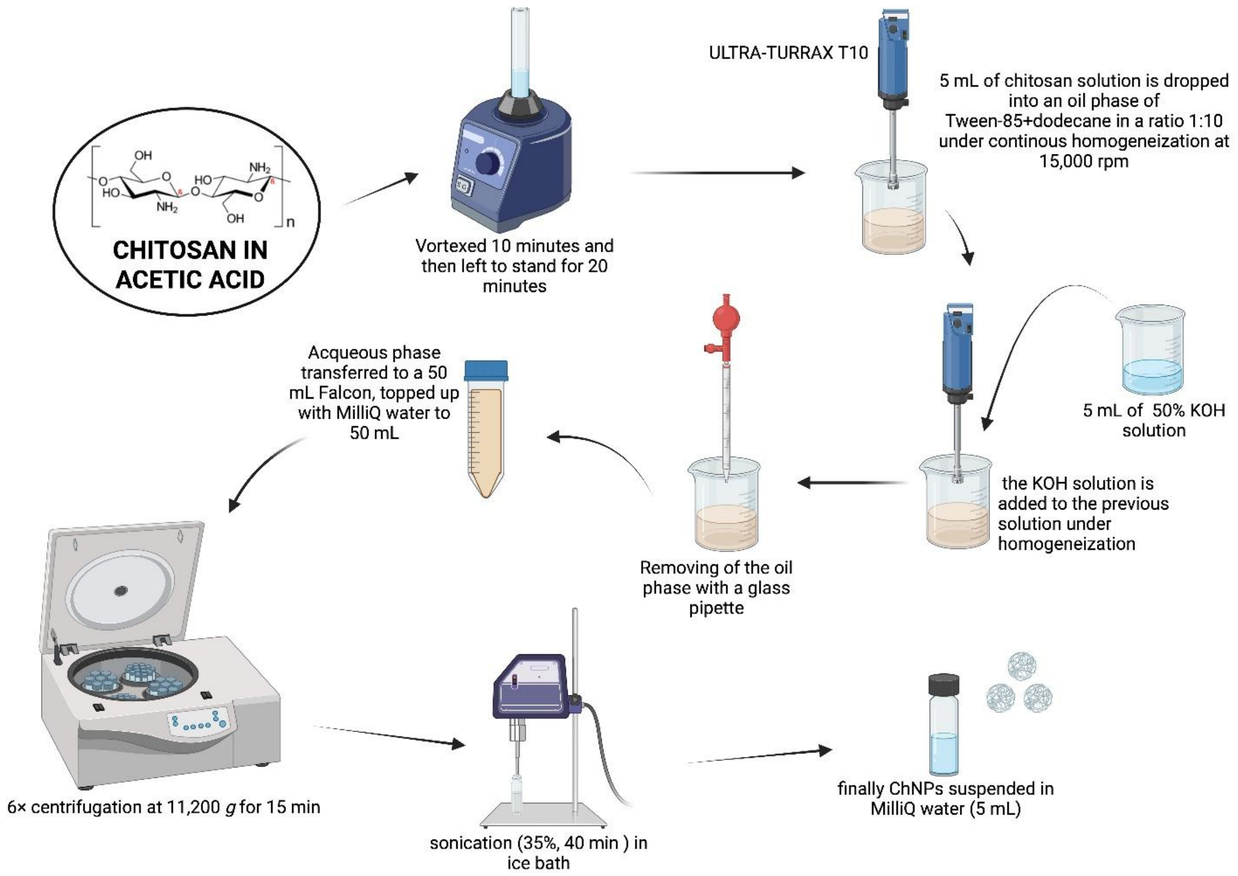

Figure S1. Scheme of the preparation of the ChNPs. Created with Biorender.

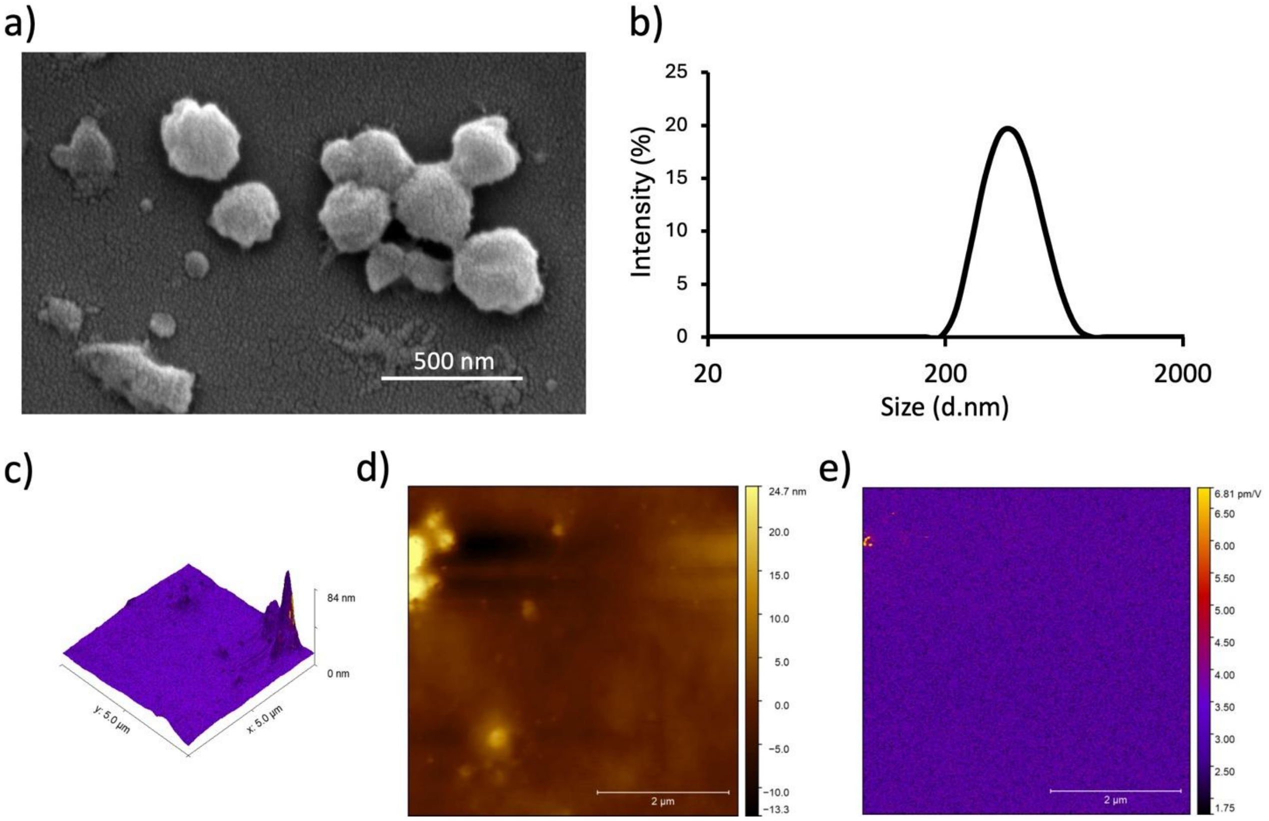

Figure S2. Characterization of ChNPs* fabricated *via* ionotropic gelation. a) SEM imaging, b) size distribution (hydrodynamic diameter), c) 3D topographic map of the ChNPs* in AFM mode (without voltage application), d) 2D topographic map in AFM mode of the same scanned area, e) PFM of the of the same scanned area.

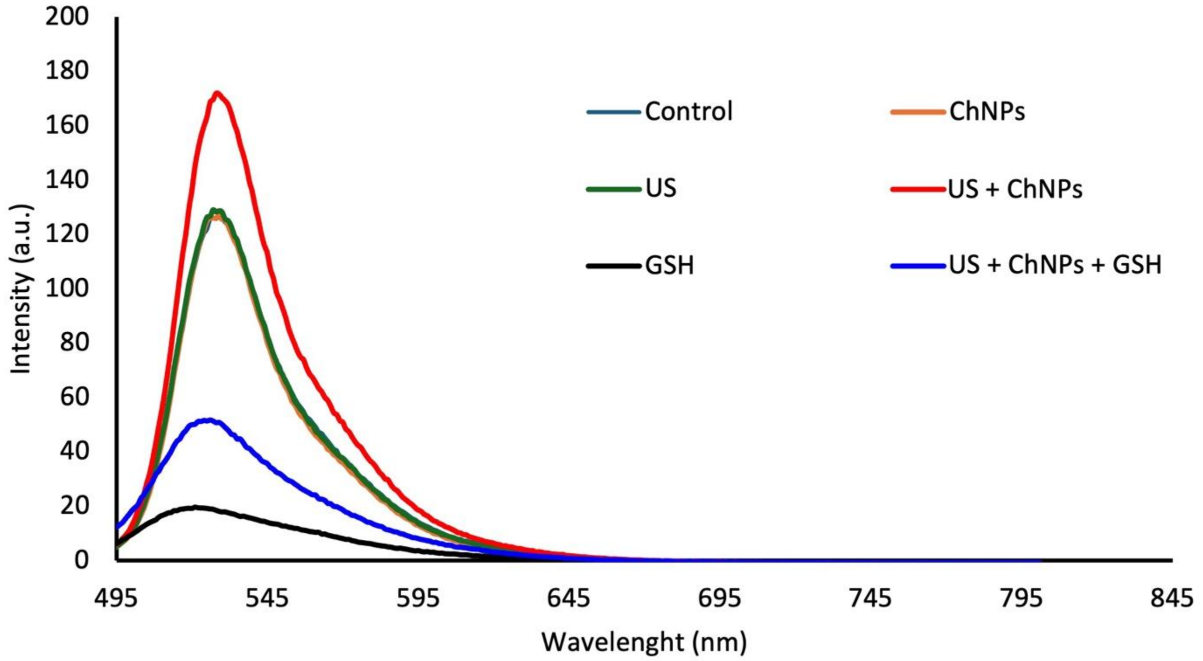

Figure S3. Representative fluorescence emission spectra of Singlet Oxygen Sensor Green, reporting $^1O_2$ generation in different experimental conditions.

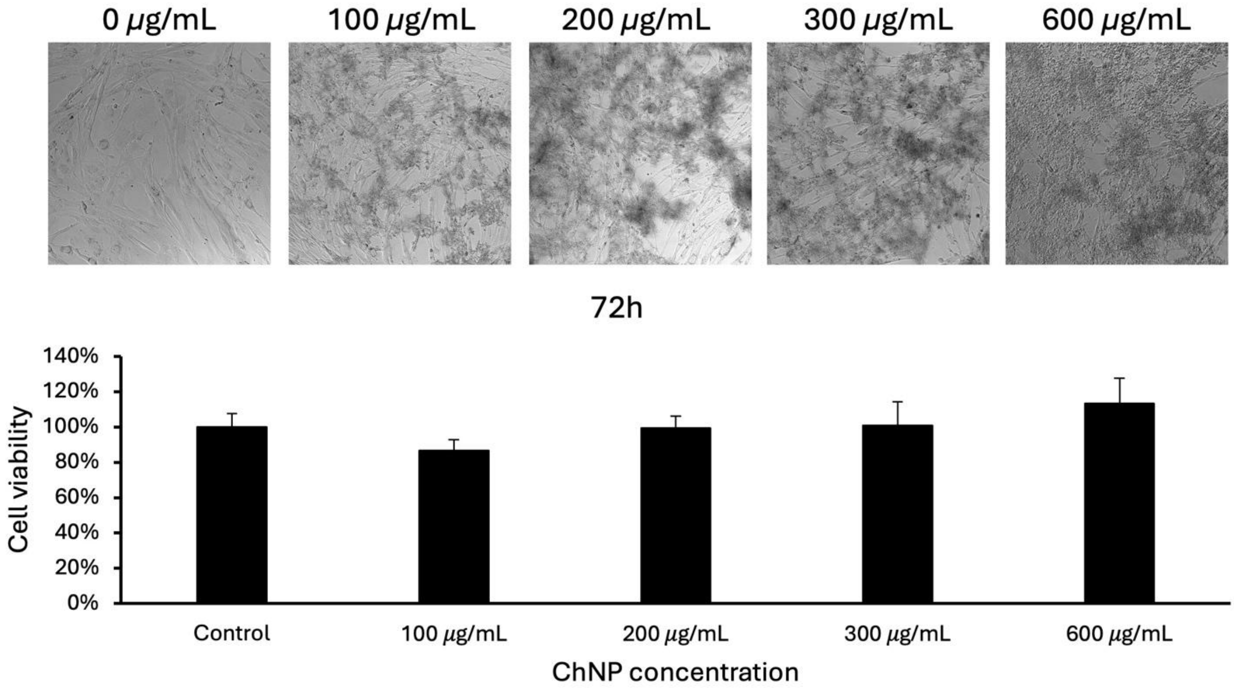

Figure S4. Cytocompatibility of ChNPs on patient-derived GBM cells. a) Representative optical microscopy images of the cells at 24 h treatment with ChNPs and b) WST-1 assay at 72 h.

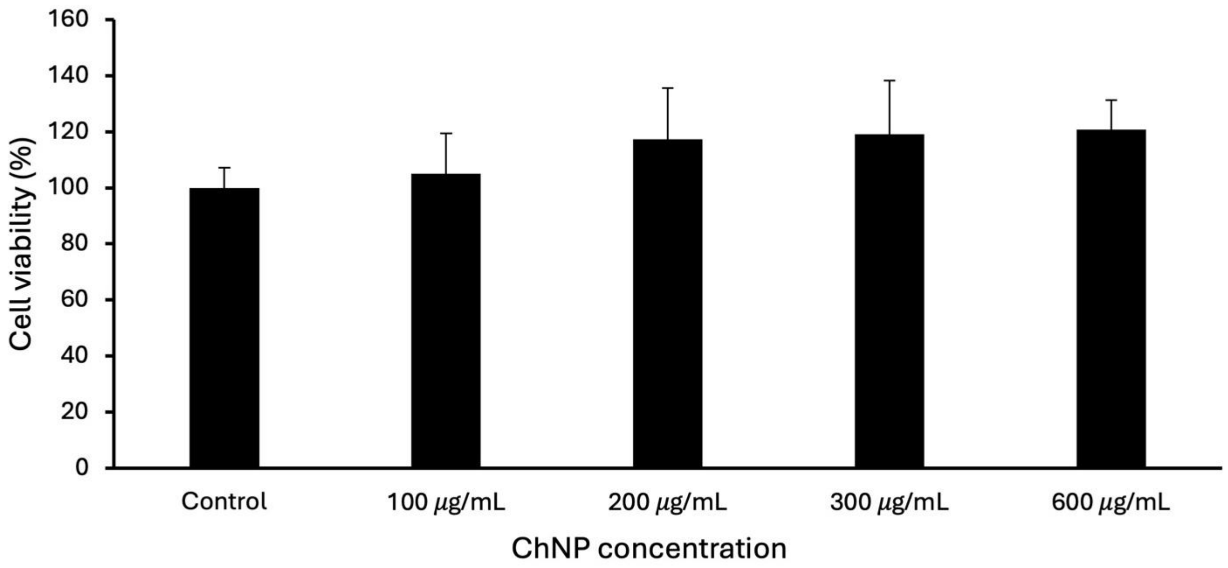

Figure S5. WST-1 cytocompatibility assay on healthy human astrocytes at 72 h of treatment with ChNPs.

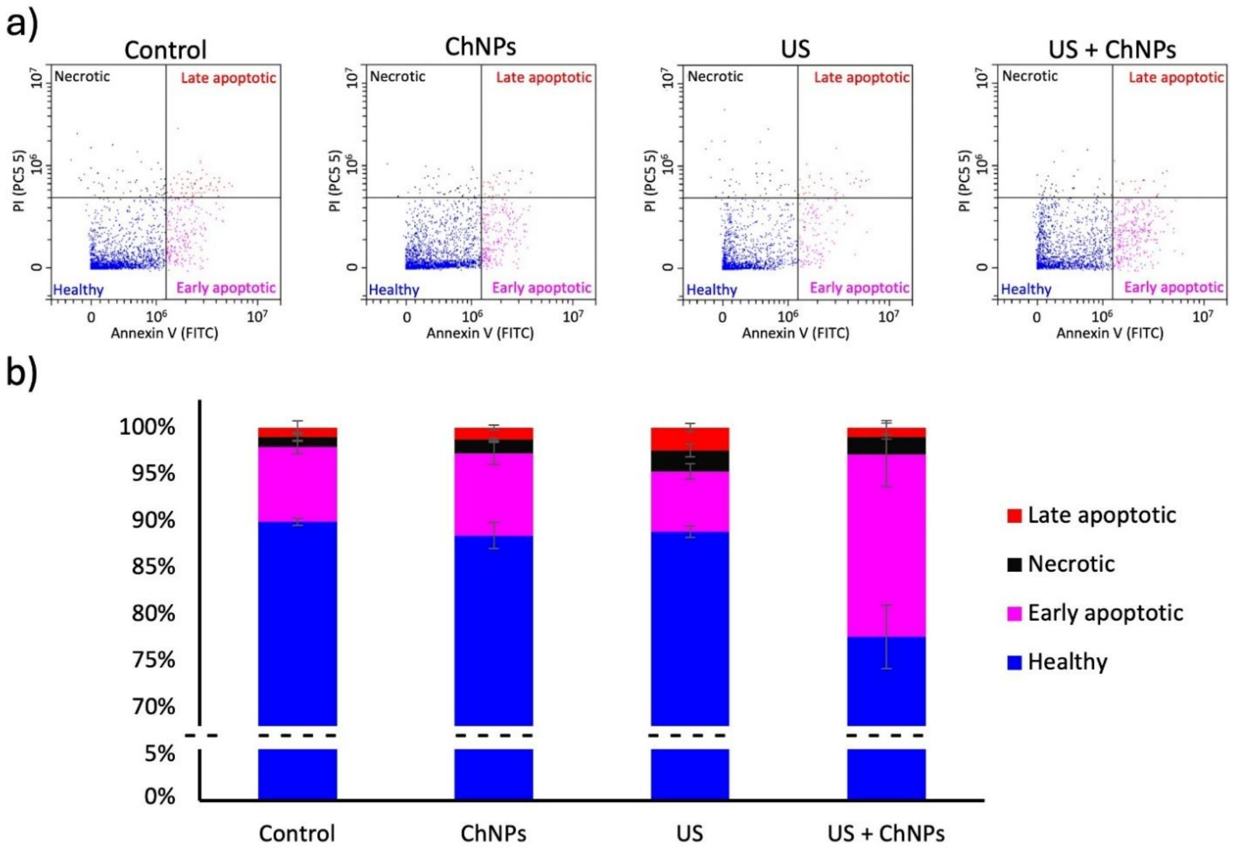

Figure S6. a) Representative annexin flow cytometry plots of V–FITC/PI staining performed on GBM patient-derived spheroids under Control, ChNPs, US, and US + ChNPs conditions, showing healthy (annexin V^{-}/PI^{-}), early apoptotic (annexin V^{+}/PI^{-}), late apoptotic (annexin V^{+}/PI^{+}), and necrotic (annexin V^{-}/PI^{+}) cell populations. b) Quantification of healthy (blue), early apoptotic (magenta), late apoptotic (red), and necrotic (black) cells (mean ± standard deviation).

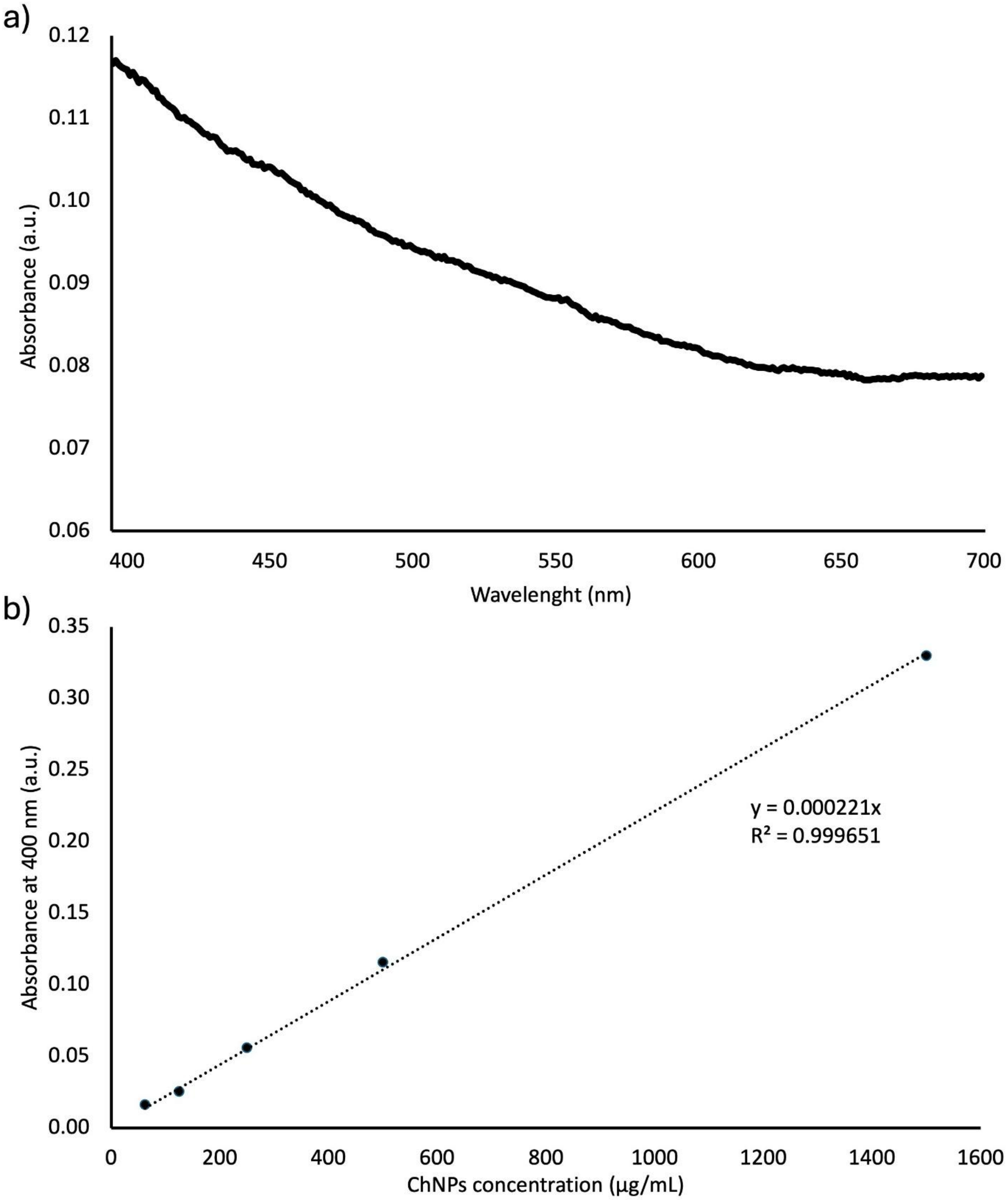

Figure S7. a) UV-Vis absorption spectrum of ChNPs at a concentration of 500 µg/mL, acquired in the wavelength range of 400–700 nm (measurements were performed in PBS buffer at pH 7.4); b) calibration curve correlating the concentration of ChNPs with their absorbance at 400 nm (R^2 = 0.9997).
